# The Impact of Maternal Obesity on Offspring Cardiovascular Health: A Systematic Literature Review

**DOI:** 10.3389/fendo.2022.868441

**Published:** 2022-05-20

**Authors:** Lois Kankowski, Maddalena Ardissino, Celeste McCracken, Adam J. Lewandowski, Paul Leeson, Stefan Neubauer, Nicholas C. Harvey, Steffen E. Petersen, Zahra Raisi-Estabragh

**Affiliations:** ^1^ Barts and the London School of Medicine and Dentistry, London, United Kingdom; ^2^ Imperial College School of Medicine, Imperial College London, United Kingdom; ^3^ Nuffield Department of Population Health, University of Oxford, Oxford, United Kingdom; ^4^ Division of Cardiovascular Medicine, Radcliffe Department of Medicine, University of Oxford, Oxford, United Kingdom; ^5^ National Institute for Health Research Oxford Biomedical Research Centre, Oxford University Hospitals NHS Foundation Trust, Oxford, United Kingdom; ^6^ MRC Lifecourse Epidemiology Centre, University of Southampton, Southampton, United Kingdom; ^7^ NIHR Southampton Biomedical Research Centre, University of Southampton and University Hospital Southampton NHS Foundation Trust, Southampton, United Kingdom; ^8^ William Harvey Research Institute, NIHR Barts Biomedical Research Centre, Queen Mary University of London, London, United Kingdom; ^9^ Barts Heart Centre, St Bartholomew’s Hospital, Barts Health NHS Trust, West Smithfield, United Kingdom

**Keywords:** obesity, maternal obesity, women’s health, cardiovascular disease, congenital heart disease, cardiometabolic disease, lifecourse epidemiology

## Abstract

**Objective:**

Obesity and cardiovascular disease are major global public health problems. Maternal obesity has been linked to multiple adverse health consequences for both mother and baby. Obesity during pregnancy may adversely alter the intrauterine environment, which has been hypothesised to predispose the offspring to poorer cardiovascular health throughout life. In this paper, we systematically review current literature examining the links between maternal obesity and offspring cardiovascular health.

**Methods:**

This study is registered with PROSPERO (CRD42021278567) and was conducted in accordance with the PRISMA guidelines. A comprehensive systematic literature search was conducted, including two electronic databases (Ovid Medline, Embase), cross-referencing, author searching, and grey literature searches. We selected studies exploring the relationship between maternal obesity and offspring cardiovascular health, using pre-defined eligibility criteria. Studies were critically appraised using the ROBINS-I tool.

**Results:**

From 1,214 results, 27 articles met the eligibility criteria. Multiple cardiovascular outcomes were considered, including congenital heart disease, cardiometabolic parameters, and cardiovascular diseases in neonates, children, and adults. In these studies, maternal obesity was consistently associated with congenital heart disease, several adverse cardiometabolic parameters throughout life including higher body mass index and insulin levels, and greater risk of cardiovascular disease in adulthood. Hypothesized underlying mechanisms are complex and multifactorial comprising genetic, environmental, and socioeconomic components, which can be difficult to quantify. Heterogeneity in study designs, highly selected study samples, and high risk of bias in some studies limit conclusions regarding causality.

**Conclusions:**

We identified consistent evidence of links between maternal obesity and poorer offspring cardiovascular health throughout the lifecourse, extending from the neonatal period into adulthood. Although underlying mechanisms are unclear, our findings support consideration of targeted maternal obesity prevention for promotion of offspring cardiovascular health. This all-encompassing systematic review provides critical appraisal of the latest evidence, defines gaps and biases of existing literature, and may inform potential new public health strategies for cardiovascular disease prevention.

**Systematic Review Registration:**

[https://www.crd.york.ac.uk/prospero], identifier PROSPERO (CRD42021278567).

## Introduction

The World Health Organisation (WHO) has characterised the rising prevalence of obesity as an epidemic. As a risk factor for a multitude of diseases, obesity poses a major global public health challenge (1).

The prevalence of obesity in women of reproductive age is increasing alongside that of the general population (2). Current estimates suggest that 30% of reproductive age women in the UK are obese (3). Maternal obesity is a risk factor for numerous pregnancy complications, including miscarriage, pre-eclampsia, and gestational diabetes mellitus (GDM). Obesity is also a major risk factor for the leading causes of maternal death: cardiovascular disease (CVD) and pulmonary embolism (4, 5). Indeed, between 2015-2017, almost 60% of the women who died within the first 6 weeks post-partum were overweight or obese (5). Maternal obesity also increases the risk of still-birth and neonatal mortality and is associated with significantly greater risk of foetal macrosomia, intra-uterine growth restriction, and congenital structural abnormalities (2).

Increasing evidence suggests that the adverse health consequences of maternal obesity for the offspring may extend beyond pregnancy throughout the entire life course (6, 7). Several studies have linked maternal obesity with greater likelihood of congenital heart disease (CHD) (8, 9), adverse cardiometabolic profile in childhood (10–13), and higher risk of CVD in adulthood (14). Whilst some studies have suggested that the period *in-utero* is a critical determinant of long-term offspring health (6, 7), others have not corroborated these findings (14–16). Another key consideration is the influence of shared home lifestyle on the mother-child cardiovascular risk profile. Indeed, a shared obesogenic diet and similar attitudes to physical exercise can importantly influence cardiovascular health of both mother and child, with such behavioural traits persisting into adulthood (17, 18). Furthermore, the genetic inheritance of obesogenic traits is likely to play an additionally important role in driving poorer cardiovascular health in the offspring of mothers with obesity. These factors are complex and intimately intertwined. Greater understanding of the relationship between maternal obesity and offspring cardiovascular health may provide insight into potential disease mechanisms and may inform targeted population-level CVD prevention strategies.

With the increasing rates of maternal obesity and the rising global burden of CVD, examining the relationships between maternal obesity and offspring life-long cardiovascular health is a public health priority. Research in this area faces unique challenges with multiple potential sources of bias. Previous reviews of literature in this area have limited to specific cardiovascular outcomes and life-stages, and as such do not capture the entire spectrum of cardiovascular consequences that may be related to maternal obesity. The aim of this paper is to provide an all-encompassing systematic review and critical appraisal of the literature exploring the impact of maternal obesity on offspring cardiovascular health across the entire life course.

## Methods

This systematic review was performed according to the Preferred Reporting Items for Systemic Reviews and Meta-analyses (PRISMA) protocol. Prior to conducting the review, the study was registered on the International Prospective Register of Systematic Reviews (PROSPERO; https://www.crd.york.ac.uk/prospero/; Registration Number: CRD42021278567) (19). Methods are aligned with the PRISMA statement (Transparent Reporting of Systematic Reviews and Meta-Analyses) (20) and the PRISMA checklist is provided in **Supplementary Table 1**.

### Eligibility Criteria

We set a broad remit for the review with pre-defined study inclusion criteria (**Table 1**). We included studies with any measure or estimate of maternal obesity set as the exposure of interest, and the outcome as any measures of offspring cardiovascular health at any age. We included studies of both adult and paediatric populations in the English language.

**Table 1:** Selection criteria for assessment of study eligibility.

	Inclusion Criteria	Exclusion Criteria
**Exposure**	Maternal obesity – pre-pregnancy, during pregnancy. Excessive gestational weight gain	Primary focus of the study on other maternal conditions e.g. gestational diabetes, hypertensive disorders of pregnancy. Maternal BMI or other objective measure of obesity not recorded before or during pregnancy
**Outcome**	Offspring cardiovascular health – any cardiovascular disease outcome at any age (paediatric or adult) accepted Surrogate markers of offspring cardiovascular health e.g. blood pressure	Focus on other aspects of offspring health not relating to cardiovascular health Primary focus of study on maternal health
**Time frame**	Any time frame	
**Study Design**	Original research study Quantitative studies Qualitative studies Human studies	Reviews* Systematic Reviews* Meta-analyses* Case Reports Opinion Papers Animal studies
**Availability**	Able to access full text PDF** English language	Non-English language

*these papers were screened for relevant original research papers as part of our “cross-reference” searches. **As per institutional access of authors.

### Search Strategy

The searches were carried out by two independent investigators (LK, MA) using the Ovid Medline (1946-October 2020) and EMBASE electronic databases. In Ovid Medline, we used the advanced search option to select relevant MeSH (Medical Subject Headings) terms. To capture broad results, we used the explode function, included all subheadings, and included a keyword search for each term using the multi-purpose (mp) function. Search terms were combined using Boolean operators. The results were limited to English language articles and human studies (**Table 2**). The titles and abstracts of the search results were visually screened for eligibility according to pre-defined criteria by LK and MA (**Table 1**). A similar approach was taken to searching the Embase database to identify further unique hits. Additional studies were sought through cross-referencing, author searches, and grey-literature searches. The full text of selected papers was then examined to confirm eligibility. Papers that did not meet these criteria were excluded. In cases of ambiguity, eligibility for inclusion was determined after full text review and discussion with project supervisors (ZRE, SEP).

**Table 2:** Search terms and combinations used for EMBASE and Ovid: Medline searches.

	Search Terms
EMBASE	(Maternal Obesity OR (Mothers AND (Obesity OR Body Mass)) AND (Cardiovascular Disease OR Heart Disease)
Ovid: Medline	(Obesity, Maternal OR (Mothers AND (Overweight OR Body Mass Index) AND (Cardiovascular Diseases OR Heart Diseases)

The search terms presented comprise Medical Subject Headings (MeSH) for Medline and, the equivalent Emtree for EMBASE, linked using Boolean operators.

### Risk of Bias Assessment

The studies were assessed for their risk of bias using the ROBINS-I (Risk of Bias in Non-randomised Studies – of Interventions) tool (21). This tool assesses the risk of bias in seven domains by asking a series of questions, before coming to an overall judgement about risk of bias (low, moderate, serious, critical). The seven domains comprise potential bias from confounding, participant selection, intervention (exposure) classification, deviations from intended intervention (exposure), missing data, outcome measurement, and selective reporting of results. Further explanations of these domains and criteria for assessment of overall risk of bias are available in a dedicated publication (21).

## Results

**Figure 1:**
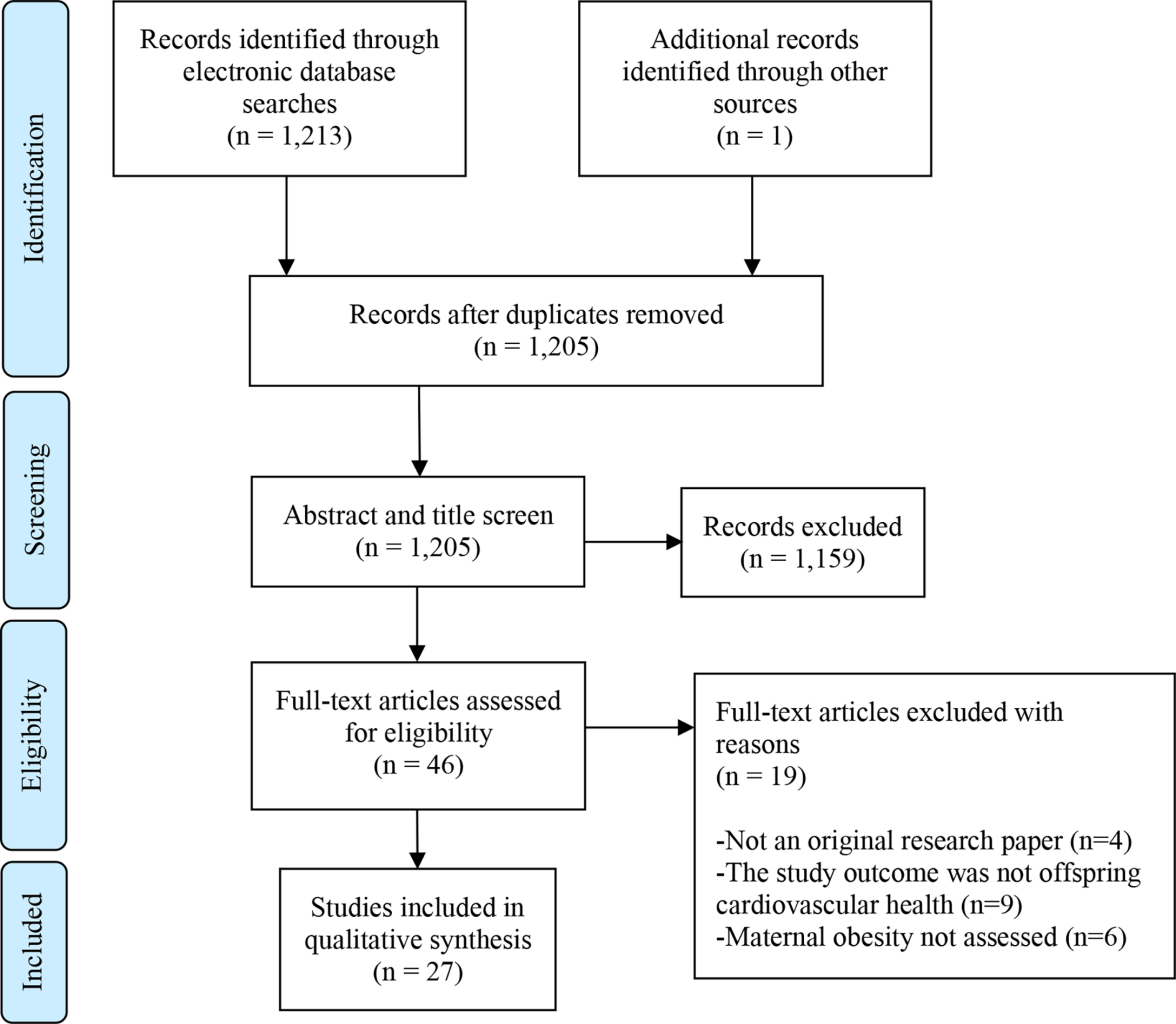
PRISMA Flow Diagram. Flow chart as per: Moher D, Liberati A, Tetzlaff J, Altman DG, The PRISMA Group (2009). *P*referred *R*eporting *I*tems for *S*ystematic Reviews and *M*eta-*A*nalyses: The PRISMA Statement. PLoS Med 6(7): e1000097. doi: 10.1371/journal.pmed1000097.

The search generated 1,213 results, including 9 duplicates. One study was identified from unpublished literature (doctoral thesis) searching. 1,159 papers were excluded following the title and abstract screen. This left 46 articles, which were read in full. Of these, 27 studies met the eligibility criteria and are included in the review (**Figure 1**).

### Study Characteristics

The 27 included studies were published between 2011 and 2020 in 11 different countries. All were observational studies. All used Maternal pre-pregnancy body mass index (MppBMI) or maternal body mass index (BMI) during pregnancy as the main exposure of interest. Six studies used gestational weight gain (GWG) as an additional exposure. One study also used skinfold thickness and fat percentage by bioimpedance. The studies measured cardiovascular health outcomes in offspring at various ages: 10 papers studied neonates, 11 studied children (0-18years), and eight studied adults (17-86 years). A diverse range of outcomes were considered, including CHD, cardiometabolic parameters (such as, BMI, blood pressure (BP) measurement, serum lipids, and serum insulin levels), and incident/prevalent CVD.

**Figure 2:**
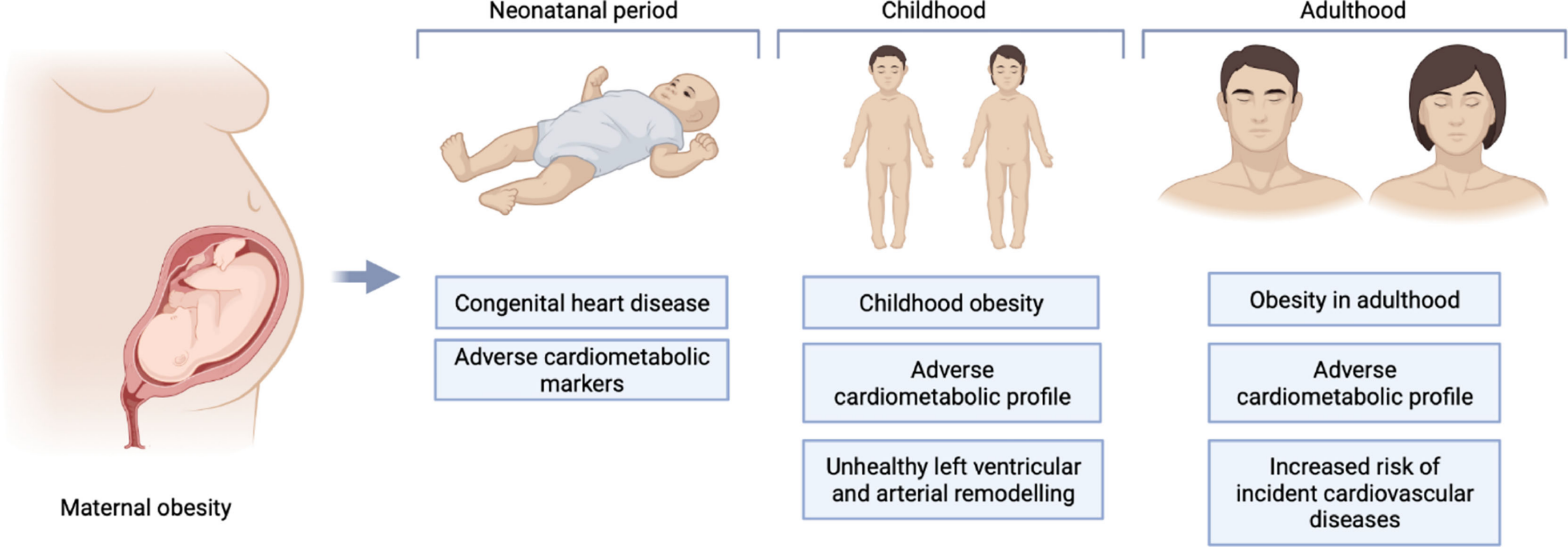
Summary of areas examined by studies included in the review.

We organised the identified studies according to the age range studied and the outcome measured: neonatal CHD (n=7), neonatal cardiometabolic parameters (n=1), childhood cardiometabolic parameters (n=11), adult cardiometabolic parameters (n=5) and adult CVD (n=3). The characteristics, outcomes, and exposures of studies are summarised in **Figure 2**, and in **Tables 3**–**7**.

**Table 3:** Characteristics of studies: Maternal obesity and neonatal congenital heart disease.

Study parameters	Exposures and outcomes	Confounders and exclusions	Summary of results	Overall bias
**Alvarado-Terrones et al., 2018** (22), Mexico **Population:** Neonates **Cases:** 55 **Controls:** 152 **Setting:** Not specified	**Exposure:** MppBMI, unclear when and how measured **Outcome:** Presence of TAPVC **Ascertainment of outcome:** Echocardiography	**Confounders:** Maternal age, sex of neonate, parity. **Exclusions:** Maternal history of addiction, pre-eclampsia, or diabetes (type1, 2, or gestational).	Pre-pregnancy obesity is associated with increased risk of TAPVC in offspring; compared with normal/ideal weight mothers: overweight [OR: 1.9 (0.9, 3.8)] obese [OR: 3.7 (1.5, 9.5)]	Moderate
**Tang et al., 2015** (23), USA **Population**: Neonates **Cases:** 569 **Controls:** 1,644 **Setting:** National Birth Defects Prevention Study. Cases and controls were identified retrospectively through medical records from 19 hospitals.	**Exposure:** MppBMI (self-report, unclear when measured) and several SNPs *via* blood or buccal sample genotyping. **Outcome:** Presence of OHDs. **Ascertainment of outcome:** Medical records (echocardiography, surgical reports, cardiac catherizations reports, autopsies)	**Confounders:** Folic acid intake during pregnancy, maternal ethnicity. **Exclusions:** Cases where pregnancy was affected by a known single gene disorder, chromosomal abnormality or syndrome.	Pre-pregnancy obesity is associated with increased risk of OHD in offspring: overweight [RR: 1.27 (1.07, 1.51)] obese [RR: 1.37 (1.15, 1.63)] Identified 19 maternal SNPs and 33 infant SNPs that interacted with maternal obesity to increase the risk of OHDs. Of these, there were 10 SNPs that were protective in normal weight women, but adverse expression in obese women.	Critical
**Brite et al., 2014** (24), USA **Population:** Neonates **Cases:** 1,388 **Controls:** 120,427 **Setting:** Consortium on Safe Labor prospective cohort study, across 19 hospitals	**Exposure:** MppBMI (medical records) **Outcome:** Presence of CHD **Ascertainment of outcome:** Discharge records	**Confounders:** Hospital site, age, race, insurance, maternal smoking. In a subset of 5,131 pairs, further adjustment was made for abnormal maternal glucose tolerance. **Exclusions:** Multiples gestations, mothers with pregestational diabetes. Infants with aneuploidy.	Pre-pregnancy obesity is associated with increased risk of CHD in offspring: overweight [OR: 1.15 (1.01, 1.32)]) obese [OR: 1.26 (1.09, 1.44)] significant trend effect (increasing risk with BMI) Increased risk for specific types of CHD with maternal obesity: conotruncal defects [OR: 1.33 (1.03, 1.72)] atrial septal defects [OR: 1.22 (1.04, 1.43)] ventricular septal defects [OR = 1.38 (1.06, 1.79)] In a subset of 5,131 pairs, obesity was still associated with increased risk of CHD after adjusting for maternal glucose tolerance.	Moderate
**Ghaderian et al., 2014** (25), Iran **Population:** Neonates **Cases:** 164 **Controls:** 158 **Setting:** Admissions to cardiology during 2011-2012. Controls were matched by region of birth, place of residence and age.	**Exposure:** MppBMI (medical records, questionnaire) **Outcome:** Presence of CHD **Ascertainment of outcome:** Established diagnosis of CHD by echocardiography, angiography, or other appropriate methods.	**Confounders:** None **Exclusions:** Family history of CHD, eclampsia, exposure to cardiac teratogens, substance abuse or radiation, mothers with gestational diabetes or pre-existing diabetes. Infants with genetic syndromes.	No association between pre-pregnancy obesity and risk of CHD in confounder-adjusted models: Overweight [OR: 0.98 (0.31, 3.10)] and obese women [OR: 1.16 (0.34, 4.00)] were not more likely to have an infant with a congenital heart defect.	Critical
**Madsen et al., 2013** (26), USA **Population:** Neonates **Cases:** 11,263 **Controls:** 140,470 **Setting:** Cases were identified through birth-hospital discharge records in the Comprehensive Hospital Abstract Reporting System (CHARS). Controls were randomly selected and matched on year of delivery.	**Exposure:** MppBMI (maternal pre-natal record, hospital medical record, or self-report if neither available). For deliveries prior to 2003, height was not recorded on birth certificate so was taken from driver’s license records. **Outcome:** Presence of CHD **Ascertainment of outcome:** Birth-hospital discharge records.	**Confounders:** Gestational diabetes **Exclusions**: Multiple births, preterm births with patent ductus arteriosus, all infants with potentially CHD-associated chromosomal abnormalities, women with existing diabetes.	Pre-pregnancy obesity is associated with increased risk of CHD in offspring [OR: 1.22 (1.15, 1.3)]. The strength of the association increased with increasing BMI [e.g., BMI 40+: OR: 1.49 (1.32, 1.69)] MppBMI is associated with significantly higher risk of several subtypes: Left ventricular outflow tract defects [OR: 1.27 (1.02, 1.59)] Right ventricular outflow tract defects [OR: 1.43 (1.2, 1.69)] Hypoplastic left heart syndrome [OR: 1.86 (1.13, 3.05)]. There was no association with CTDs [OR: 1.04 (0.82, 1.33)].	Moderate
**Dolk et al., 2020** (27), UK **Population:** Neonates **Cases:** 242 **Controls:** 966 **Setting:** The Baby Hearts Study **-** Northern Ireland Paediatic Cardiology Centre.	**Exposure:** MppBMI - recorded by midwife at booking (10-12weeks gestation). **Outcome:** Presence of CHD **Ascertainment of outcome:** Cases were classified by paediatric cardiologists based on clinical records and International Paediaric Cardiology Code criteria.	**Confounders:** Adjusted for maternal age, previous pregnancy, maternal education, socioeconomic deprivation area of residence, folic acid supplementation, smoking, antidepressant prescription in first trimester, dietary class, pregnancy stress, multiple stressors. **Exclusions:** Mothers with pre-gestational diabetes. Infants with genetic syndromes.	Compared with normal BMI mothers, obesity, based on BMI at booking, was not associated with CHD risk, before or after adjustment for diabetes and other covariates for any obesity category: Overweight [OR: 1.07 (0.76, 1.48); aOR: 1.02 (0.71, 1.46)] Obese [OR: 1.00 (0.67-1.48); aOR: 0.98 (0.63-1.51)] Morbidly obese [OR: 0.86 (0.37-1.99); aOR: 0.70 (0.28-1.81)]	Moderate
**Kaplinski et al., 2018** (28), USA **Population:** Neonates **Cases:** 721 **Setting:** Parents of children with conotruncal defects identified *via* Children’s Hospital of Philadelphia (1992-2010) and the Paediatric Cardiac Genomic Consortium (2010-2012).	**Exposure:** Genetic risk scores for obesity (maternal and paternal) **Outcome:** Presence of CTDs **Ascertainment of outcome:** Echo/other imaging, confirmed by a paediatric cardiologist.	**Confounders:** None **Exclusions:** Non-Caucasian ethnicity. Patients with genetic syndromes were excluded.	Among parents of children with CTD, there was no significant difference in genetic obesity risk score between mothers and fathers – Obesity (30 SNPs): OR: 1.73 (0.82, 3.64) Sensitivity analysis excluding mothers with, pre-eclampsia, hypertension and pregestational diabetes yielded similar results.	Severe

aOR, adjusted odds ratio; CHD, Congenital heart disease; CTDs, conotruncal defects; MppBMI, Maternal pre-pregnancy body mass index; OHD, obstructive heart defects; OR, odds ratio; RR, Risk ratio; SNPs, Single Nucleotide Polymorphisms; TAPVC, total anomalous pulmonary venous connection.

**Table 4:** Characteristics of the studies: Maternal obesity and neonatal cardiometabolic parameters.

Study parameters	Exposures and outcomes	Confounders and exclusions	Summary of results	Overall bias
**Lemas et al., 2015** (29), USA **Population:** Neonates (within 72hrs) **Cases:** 753 **Controls:** 1,012 **Setting: The Healthy Start study.** Pregnant women were recruited from the University hospital obstetric clinics from 2009-2014.	**Exposure:** MppBMI (medical records 90%, self-report 10%). Gestational weight gain - difference between pre-pregnant weight and last weight before delivery. **Outcome:** Neonatal cardio-metabolic markers including; Cord blood glucose, insulin, glucose-insulin ratio, total cholesterol, HDL-c, TG, free fatty acids, leptin. **Ascertainment of outcome:** Sampling and biochemical assays	**Confounders:** Child gestational age and sex, maternal age, gravidity, race/ethnicity, smoking in pregnancy, household income, infant birth weight/infant fat mass **Exclusions**: Women expecting multiple births, previous preterm (<25 wks) birth or stillbirth, pre-existing diabetes, asthma managed with steroids, cancer or serious psychiatric illness.	After adjusting for infant fat mass, higher pre-pregnancy BMI is significantly associated with: Lower HDL-c [B: -0.11 (-0.21, -0.02)] Higher leptin [beta = 0.51 (0.33, 0.69)] There is borderline evidence for higher insulin and lower glucose/insulin ratio with higher MppBMI.	Moderate

HDL-c, high-density lipoprotein cholesterol; MppBMI, Maternal pre-pregnancy body mass index; TG, triglyceride.

**Table 5:** Characteristics of the studies: Maternal obesity and childhood cardiometabolic parameters.

Study parameters	Exposures and outcomes	Confounders and exclusions	Summary of results	Overall bias
**Cox et al., 2020** (30), Belgium **Population:** Children 4-6 years **Cohort size:** 240 **Setting:** Mother-newborn pairs were recruited from Environmental Influence on Early Ageing birth cohort study. Recruited at birth.	**Exposure:** MppBMI based on measurements at first antenatal visit (7-9 weeks gestation). **Outcome:** Child’s BMI, waist circumference, SBP, DBP, mean arterial pressure. CRAE, CRVE, AVR, TI. **Ascertainment of outcome:** Anthropometric measurements, automated BP measurement, analysis of retinal images.	**Confounders:** Sex, gestational age, parity, newborn race/ethnicity, maternal age, maternal education, maternal smoking, gestational weight gain, date of follow up, season of follow-up, age of the child at follow-up, child’s birth weight and current BMI.	In confounder-adjusted models, higher MppBMI (per 1 kg/m^2^) is associated with higher values in offspring for: BMI (kg/m^2^): 0.08 (0.04, 0.12) Waist circumference (cm): 0.14 (0.03, 0.25) SBP (mmHg): 0.27 (0.03, 0.51) DBP (mmHg): 0.26 (0.08, 0.45) Mean arterial pressure (mmHg): 0.26 (0.08, 0.44) TI (x10^3^) 0.40 [0.01, 0.80] In adjusted models, there was no significant association between pre-pregnancy BMI and: CRAE [-0.18 (-0.55, 0.18)] CRVE [0.31 (-0.17, 0.79)] AVR [-0.10 (-0.26, 0.06)]. Paternal BMI was not associated with blood pressure of offspring, suggesting direct intrauterine (rather than shared familial) mechanisms.	Severe
**Veena et al., 2013** (31), India **Population:** Children 9.5 years **Cohort size:** 504 **Setting:** The Mysore Parthenon study. Women were recruited at antenatal clinics at Holdsworth Memorial Hospital.	**Exposure:** Maternal adiposity by sum of skinfold thickness measurements (SS) during pregnancy - measured at 30+/- 2weeks. Paternal adiposity - measured at 5 years. **Outcome:** Anthropometry, fat percentage, plasma glucose, insulin, LDL, HDL, TAG concentrations and BP. **Ascertainment of outcome:** BMI, WC, SS, Fat % was measured by bio-impedance. Manual BP measurement. Blood sampling.	**Confounders: C**hild’s sex and age, SES, maternal age, parity, breast-feeding duration, maternal and paternal adiposity mutually adjusted, maternal glucose concentrations during pregnancy. Further adjustment by child’s current sum of skinfold thickness. **Excluded:** GDM	Both maternal and paternal adiposity associate with cardiometabolic features of children, but these effects are largely mediated by the child’s adiposity. Higher **maternal skinfold** measures were associated with higher BMI, sum of skinfold, fat percentage, waist circumference and lower TAG concentrations in offspring. Higher **paternal skinfold** measures were associated with higher BMI, sum of skinfold, waist circumference, fasting insulin and HOMA-IR in offspring. After adjustment for child’s current sum of skinfold, only the effect of maternal skinfold on lower offspring TAG remained significant.	Moderate
**Toemen et al., 2016** (32), The Netherlands **Populations:** Children 6 years **Cohort size:** 4,852 **Setting:** Generation R study, studying fetal life onwards in Rotterdam.	**Exposure:** MppBMI (self-report questionnaire given at 13weeks), early 13weeks, mid 20weeks and late 30weeks (measured). **Outcome:** LVM, LVMi, relative wall thickness, aortic root diameter, fractional shortening, eccentric LVH, concentric remodelling. **Ascertainment of outcome:** Echocardiography	**Confounders: C**hild age and sex, maternal age, education, folic acid, alcohol, paternal age, first trimester calorie intake, ethnicity. Additional adjustment for child’s current BMI. **Exclusions:** Multiple births, children with cardiac abnormalities.	In confounder-adjusted models, higher MppBMI is associated with (SD change): Greater LVM [B: 0.10 (0.08, 0.13)] Greater LVMi [B:0.06 (0.03, 0.09)] Aortic root diameter [B: 0.09 (0.06, 0.12)]. However, these effects were fully explained/mediated by child’s BMI at 6 years.	Moderate
**Gaillard et al., 2015** (33), The Netherlands **Population:** Children 6 years **Cohort size:** 5,908 **Setting:** Generation R study, studying fetal life onwards in Rotterdam.	**Exposure:** MppBMI (self-report questionnaire given at 13weeks and early 13weeks; measured at mid 20weeks and late 30weeks). **Outcome:** BMI, total body and abdominal fat distribution, BP and blood levels of lipids, insulin and c-peptide. **Ascertainment of outcome:** Anthropomorphic measurements, body fat by dual-energy x-ray absorptiometry, multiple automated BP measurements, blood sampling.	**Confounders:** Child sex and age at outcome measurements, gestational age at maternal weight measurement, maternal age, educational level, ethnicity, parity, height at intake, smoking during pregnancy, alcohol consumption during pregnancy and folic acid supplement use, total calorie intake during pregnancy, delivery mode, breastfeeding duration, timing of introduction of solid foods and child’s average duration of tv-watching. Further adjustment by child’s current BMI.	In confounder-adjusted models, higher MppBMI is associated with greater BMI, SBP, HDL, insulin, and all body fat measures in offspring at 6 years. However, these effects were largely mediated by the addition of child’s BMI at 6 years. In confounder-adjusted models, GWG in early and mid-pregnancy are associated with higher offspring BMI early pregnancy (std beta 0.06 [0.04, 0.09]) mid pregnancy (std beta 0.03 [0.01, 0.05]) Other than this, GWG had no association with childhood outcomes (including fat mass, BP, lipids or insulin)	Severe
**Filler et al., 2011** (34), UK **Population:** Children 2-18 years **Cohort size:** 3,204 **Setting:** Prospective cohort study of patients attending the Children’s Hospital, London Health Science Centre were recruited from the emergency department and large outpatient clinics.	**Exposure:** MppBMI (self-report questionnaire), birthweight (questionnaire), gestational age (questionnaire), child BMI-z score (measured). **Outcome:** BP **Ascertainment of outcome:** Random BP measurements using a standardized protocol were taken at the clinic visits using automated oscillometric BP machines. BMI measured using high-precision industrial scales.	**Confounders:** None considered	In age-adjusted correlation analyses, higher MppBMI was associated with higher SBP (r=0.090, p<0.0001), higher DBP (r=0.062, p=0.0007) and higher BMI (r=0.202, p<0.0001) in offspring.	Critical
**Techur-Pedro 2015** (35), USA **Population:** Children 4-6 years **Cohort size**: 441 **Setting:** Retrospective longitudinal cohort study of live births registered on the Palau Ministry of Health Maternal and Child Health Birth Registry	**Exposure:** MppBMI (self-report) **Outcome:** Offspring BMI **Ascertainment of outcome:** BMI-z score	**Confounders:** Maternal age, self-reported maternal height, parity, tobacco use during pregnancy, anaemia, gestational weight gain Exclusions: Multiple births, pre-term births	(Chapter 4: Study 3) Among children age 4-6yr, the risk of overweight or obesity was 2.57 times higher among those born to mothers with pre-pregnancy obesity compared to the risk in children 4-6yr born to mothers with normal pre-pregnancy BMI.	Critical
**Litwin et al., 2020** (36), Finland **Population:** Children 6 years **Cohort size:** 201 **Setting:** Longitudinal observational follow-up study from Finnish Gestational Diabetes Prevention Study (RADIEL).	**Exposure:** MppBMI (self report or measured) GDM – assessed *via* Oral Glucose Tolerance Test (OGTT), MppBMI - measured and self-report, unclear gestation. **Outcome:** Childhood anthropometrics, body composition, left ventricular function **Ascertainment of outcome:** Echocardiography.	**Confounders:** Child lean body mass, age, sex, SBP, HR, maternal lean body mass. Excluded: Children with body size exceeding the 95^th^ percentile	In confounder-adjusted models, MppBMI was not associated with offspring left ventricular diastolic and systolic function across a wide range of atrial and ventricular measures of structure and function. Among obese mothers, each additional kg/m^2^ of BMI was associated with a 0.06 (0.01, 0.011) point increase in child body fat.	Serious
**Perng et al., 2014** (37), USA **Population:** Children 6-10 years **Cohort size:** 1,090 **Setting:** Recruited from Project Viva, a prospective cohort of pregnant women and their children in the US.	**Exposure:** MppBMI (self-report), asked at enrolment. GWG (difference between last clinically measured weight prior to delivery and self-reported pre-pregnancy weight). **Outcome:** Childhood overall and central adiposity, SBP, fasting insulin, glucose, HOMA-IR, TG, leptin, adiponectin, CRP, IL-6. **Ascertainment of outcome:** Anthropometric measurements, whole-body DXA scanning, automated BP measurements, blood sampling.	**Confounders:** Child sex and age, maternal ethnicity, parity, smoking habits, father’s BMI, household income. Further adjustment by child’s total fat mass **Exclusions:** Mother-child pairs with a prenatal history of type 1 or type 2 diabetes, and pregnancies < 34 wks.	In confounder-adjusted models, each 5kg/m^2^ higher MppBMI corresponded with the following features in children: higher total fat mass 0.92kg (95% CI 0.7 - 1.14), higher BMI-for-age z-score 0.27 SD (0.21 - 0.32), higher trunk fat 0.39kg (0.29 - 0.49), larger waist circumference 2.08 cm (1.64 – 2.52) higher HOMA-IR 0.10 (0.04 – 0.16) higher leptin 0.10 ng/mL (0.04 – 0.16) higher hsCRP 0.23 mg/L (0.11 – 0.35) higher IL-6 0.09 pg/mL (0.02 – 0.15) higher SBP 0.77 mmHg (0.27 – 1.27) However, after adjustment for child’s total fat mass, only IL-6 remained borderline significant.	Moderate
**Tan et al., 2015** (38), USA **Population:** Children 8-18 years **Cohort size:** 68 **Setting:** Recruited from Children’s Nutrition Research Centre at Baylor College of Medicine (through advertisement), and from the University of Pittsburgh - participants in Prenatal Exposures and Preeclampsia Prevention project.	**Exposure:** MppBMI (medical records and self-report in 16 mothers, unclear when asked during the pregnancy). **Outcome:** Childhood body composition, abdo fat distribution, BP measurement, fasting lipids, glucose tolerance **Ascertainment of outcome:** BMI, WC, BP, DXA, CT/MRI for abdominal fat distribution, OGTT, fasting LDL, HDL, TG. C Reactive Protein (CRP), HbA1c. Plasma glucose, Insulin/Glucose ratio, Whole Body Insulin Sensitivity Index, HOMA-IR.	**Confounders:** Offspring birth weight, maternal medical condition, Tanner stage, race/ethnicity. Further adjustment by child’s percentage body fat **Exclusions:** GDM, smoking, *in-utero* infections, chromosomal abnormality, history of chronic health problems/medications that may affect growth.	In confounder-adjusted models, higher MppBMI was significantly associated with child’s BMI z-score and whole-body insulin sensitivity index (WBISI). Child’s BMI z-score remained significant after adjustment for child’s percentage body fat, while the effect for WBISI became borderline. After adjusting for ethnicity, children of obese mothers were observed to have higher SBP, higher hs-CRP, and adverse insulin and lipid profiles* compared to children of normal weight mothers, however, results adjusted by child’s percentage body fat were not presented. *effect sizes and 95% CI’s were not provided	Moderate
**Wang et al., 2017** (39), USA **Population:** Children 9 years **Cohort size:** 1,279 **Setting:** Mother-infant pairs drawn from the Boston Birth Cohort.	**Exposure:** MppBMI (self-report questionnaire 2-3days post-partum). Maternal plasma homocysteine (blood sampled 2-3 days post-partum). **Outcome:** Offspring SSBP **Ascertainment of outcome:** Manual SBP measurement	**Confounders:** Maternal age, race, education, smoking, alcohol intake, parity, perceived stress during pregnancy, hypertensive disorders, diabetes and plasma Hcy. **Exclusions**: Some exclusions based on incomplete data.	In confounder-adjusted models, pre-pregnancy obesity is significantly associated with risk of elevated SBP in offspring at 9 years (OR: 1.48 [1.12, 1.97]).	Moderate
**Brandt et al., 2014** (40), Germany **Population:** Children 8 years **Cohort size:** 422 **Setting:** All women admitted to the University of Ulm to give birth to a baby between 2000 and 2001 were recruited.	**Exposure:** MppBMI (medical records, measured at first visit to gynaecologist during pregnancy). **Outcome:** Offspring insulin concentrations, BMI **Ascertainment of outcome:** Anthropometric measurements, cord blood sampling, follow-up blood sampling.	**Confounders:** Gender, gestational age, BMI at birth, maternal and paternal BMI values at the 8year follow up. **Exclusions:** GDM, birth weight < 2000g or preterm <32 weeks.	Higher MppBMI was significantly associated with higher offspring fasting plasma insulin concentrations at age 8 (partial correlation r=0.16 with adjustment for child’s gender). GWG was not correlated with offspring fasting insulin concentration at age 8 (r=-0.05).	Moderate
**Sundholm et al., 2020** (41), Finland **Population:** Children 6 years **Cohort size:** 201 **Setting:** Women in early pregnancy with increased risk of GDM due to BMI 30+ were recruited between 2008 and 2012 into RADIEL study. This study uses a sub-cohort of the RADIEL study.	**Exposure:** MppBMI (measured prospectively during pregnancy). **Outcome:** Offspring anthropometrics, arterial morphology and stiffness. **Ascertainment of outcome:** Offspring BMI, WC, body composition, lean body mass, body fat percentage, carotid and peripheral artery ultrasound, arterial pulse wave velocity.	**Confounders:** Child sex and age, GDM - GDM diagnosis, OGTT results, HOMA-IR.	Children of obese mothers have significantly higher BMI, blood pressure and carotid IMT than the healthy population. However, in confounder-adjusted models, arterial features were associated with child’s lean body mass and not child or maternal adiposity.	Critical

AVR, arteriole-to-venule ratio BMI, body mass index; BP, blood pressure; CRAE, Central retinal arteriolar equivalent; CRP, C reactive protein; CRVE, central retinal venular equivalent; CT, computed tomography; DBP, diastolic blood pressure; DXA, bone densitometry; GDM, gestational diabetes mellitus; GWG, gestational weight gain; HbA1c, glycated haemoglobin; HDL, high density lipoprotein cholesterol; HOMA-IR, Homeostasis Model Assessment of Insulin Resistance; IL, interleukin; LDL, low density lipoprotein; LVM, Left ventricular mass; LVMi, Left ventricular mass index; LVH, Left ventricular hypertrophy; MppBMI, Maternal pre-pregnancy body mass index; OGTT, oral glucose tolerance test; OR, odds ratio; SBP, systolic blood pressure; SD, standard deviation; TG, triglyceride; SS, skin-fold thickness; TAG, lower tri-acyl-glycerol; TI, tortuosity index; WC, waist circumference; WBISI, whole-body insulin sensitivity index.

**Table 6:** Characteristics of the studies: Maternal obesity and cardiometabolic parameters in adulthood.

Study parameters	Exposures and outcomes	Confounders and exclusions	Summary of results	Overall bias
**Hochner et al., 2012** (15), Israel Population: Adult offspring 32 years Cohort size: 1,400 **Setting:** The Jerusalem Perinatal study includes a subcohort of births to residents of Jerusalem between 1974-1976.	**Exposure:** MppBMI (self-report after delivery), GWG (self-report after delivery). **Outcome:** Offspring BMI, WC, BP, insulin levels, TG **Ascertainment of outcome:** Offspring BMI, WC, BP, insulin levels, TG	**Confounders:** Ethnicity, sex. Maternal and offspring characteristics at birth - parity, mother’s age, maternal smoking during the pregnancy, socioeconomic status, mother’s education years, maternal medical condition, birth weight, gestation. Offspring characteristics at age 32 - smoking status, physical activity, education. Further adjustment for offspring BMI and WC at 32years as potential mediators. **Exclusions:** Mothers on lipid lowering, BP lowering and diabetes medication.	Higher MppBMI and GWG are strongly associated with higher offspring BMI. In confounder-adjusted models, higher MppBMI was positively associated with offspring WC, SBP, DBP, insulin, TG at 32years. However, these effects were not significant after adjusting for offspring BMI. In confounder-adjusted models, higher GWG was associated with higher offspring WC, SBP and TG, however these were also not significant after adjusting for offspring BMI.	Moderate
**Kaseva et al., 2019** (16), Finland Population: Adult offspring 23-25 years Cohort size: 906 **Setting:** Participants were from two prospective birth cohorts - ESTER maternal Pregnancy Disorders Study and the Arvo Ylppo Longitudinal Study.	**Exposure:** Pre-pregnancy overweight/obesity (unclear how measured), GDM. **Outcome:** Measured cardiometabolic biomarkers from blood, measured BP, resting HR **Ascertainment of outcome:** Fasting glucose, insulin, SHBG, HDL, LDL, TG, lipoprotein, ApoA1, ApoB, FFA, testosterone, uric acid, CRP, ALT, AST, GGT, SBP, DBP, HR.	**Confounders:** Age, sex, source cohort, gestational age, birthweight SD score, maternal hypertension or preeclampsia during pregnancy, maternal smoking during pregnancy, parental educational attainment, and parental hypertension, diabetes, stroke or myocardial infarction, BMI, height, and daily smoking.	In minimally adjusted models, offspring of obese/overweight normoglycemic mothers had higher fasting glucose and fasting serum insulin. However, these were not significant when adjusted for confounders and/or current offspring characteristics, including BMI or body fat percentage. No difference in HR, BP, SHBG, FFA, TC, HDL-C, LDL-C, TGs, lipoprotein, ApoA1, ApoB, liver test results, uric acid – similar between offspring of obese/overweight normoglycaemic mothers and control groups.	Insufficient information
**Lahti-Pulkkinen et al., 2019** (46), UK Population: Adult offspring 1-62years Cohort size: 118,201 **Setting:** Birth records in the Aberdeen Maternity and Neonatal databank linked to Scottish Care Information-Diabetes.	**Exposure:** MppBMI (recorded at first antenatal visit, medical records). **Outcome:** Any diabetes, T1DM, T2DM **Ascertainment of outcome:** Any diabetes, T1DM, or T2DM from Diabetes database.	**Confounders:** Maternal history of diabetes before pregnancy, maternal history of hypertension, maternal age at delivery, gestation when weight was measured, deprivation category, parity and sex of offspring Did not further adjust for offspring BMI although **Table 1** shows significant differences in offspring BMI between maternal obesity groups. Gestation when weight was measured, maternal DM before pregnancy, maternal history of hypertension, age at delivery, maternal history of hypertension, parity, socioeconomic status, sex of offspring.	In confounder-adjusted models, offspring of obese and overweight mothers had an increased hazard of T2DM compared with mothers with normal BMI Overweight [aOR: 1.39 (1.06, 1.83)] Obese [aOR: 3.48 (2.33, 5.06)] There was no association between maternal BMI and T1DM: Overweight [aOR: 1.16 (0.92, 1.46)] Obese [aOR: 1.25 (0.89, 1.75)]	Severe
**Eriksson et al., 2015** (45), Finland Population: Adult offspring (mean age 62 years) Cohort size: 2,003 **Setting:** Individuals from the Helsinki Birth Cohort study (born 1934-44)	**Exposure:** Maternal obesity in late pregnancy. Measured in hospital prior to delivery. **Outcome:** Offspring BMI, lean body mass, fat mass. SBP, DBP, fasting glucose, insulin concentrations, blood lipids, inflammatory markers, adipocytokines. **Ascertainment of outcome:** Body measurements, fat mass by bio-impedance, manual BP, blood sampling.	**Confounders:** Age and sex.	Adjusting for age, higher maternal BMI was associated with significantly higher BMI, lean body mass, and fat mass among offspring, and a higher body fat percentage in women. Maternal BMI was not associated with SBP or DBP, fasting glucose, insulin concentrations, blood lipids or inflammatory markers in the offspring.	Severe
**Lawrence et al., 2014** (47), Israel Population: Adult offspring (birth, 17 years, and 32 years) Cohort size: 1,400 **Setting:** Jerusalem Perinatal Study - includes a sub-cohort of all births to residents of Jerusalem between 1974-1976.	**Exposure:** MppBMI and GWG (self-report after delivery). **Outcome:** Interval change in offspring BMI **Ascertainment of outcome:** Interval change in offspring BMI from 17-32years	**Confounders:** Ethnicity and sex, characteristics at time of birth (i.e., maternal years of education and smoking, SES based on father’s occupation, birth weight, and gestational age), offspring characteristics at age 17 years (i.e., BMI at age 17 years), and off- spring characteristics at age 32 years (i.e., smoking, physical activity, caloric intake, education type, and years of education). Further adjustment with two genetic scores, one for maternal adiposity and one for offspring adiposity	In confounder-adjusted models, one SD change in MppBMI was associated with a 0.83kg/m^2^ increase in interval change in offspring BMI between time points. This was not affected by genetic scores. In confounder-adjusted models, one SD change in GWG was associated with a 0.75kg/m^2^ increase in interval change in offspring BMI. With the addition of genetic scores, this effect was substantially weakened but remained significant.	Moderate

ALT, Alanine transferase; aOR, adjusted odds ratio; ApoA1, apolipoprotein A1; ApoB, apolipoprotein B; AST, aspartate transaminase**;** BMI, body mass index; BP, blood pressure; CRP, C reactive protein; DBP, diastolic blood pressure; DM, diabetes mellitus; FFA, free fatty acids; GDM, gestational diabetes mellitus; GGT, gamma glutamyl transferase; GWG, gestational weight gain; HDL, high density lipoprotein cholesterol; HR, heart rate; LDL, low density lipoprotein cholesterol; MppBMI, Maternal pre-pregnancy body mass index; OR, odds ratio; T1DM, Type 1 diabetes mellitus; T2DM, Type 2 diabetes mellitus; TG, triglyceride; SBP, systolic blood pressure; SD, standard deviation; SHBG, sex hormone binding globuline; WC, waist circumference.

**Table 7:** Characteristics of the studies: Maternal obesity and adult cardiovascular disease.

Study parameters	Exposures and outcomes	Confounders considered	Summary of results	Overall Bias Assessment
**Reynolds et al., 2013** (14), UK **Population:** Adult offspring 34-61 years **Cohort size:** 37,709 **Setting:** Birth records from the Aberdeen Maternity and Neonatal databank linked to the General Register of Deaths, Scotland and the Scottish Morbidity Record systems. 1950-2012.	**Exposure:** BMI during pregnancy (measured at first antenatal visit, some >20weeks). **Outcome:** all-cause mortality, incident cardiovascular hospitalisations (up to 1 Jan 2012). **Ascertainment of outcome:** Hospital records	**Confounders:** Maternal age at delivery, gestation when weight was measured, social class, parity, sex of offspring, gestation at delivery, birth weight, current age of offspring.	In confounder-adjusted models, obesity during pregnancy is associated with increased risk of adverse outcomes in offspring: All-cause mortality [HR: 1.35 (1.17, 1.55)] Death before age 65 years [HR: 1.40 (1.17, 1.68)] Combined cardiovascular events [HR: 1.26 (1.06, 1.57)] Maternal overweight during pregnancy was also associated with increased risk for the same events, but with smaller point estimates (HRs: 1.11 to 1.19).	Moderate
**Eriksson et al., 2014** (48), Finland **Population**: Adult offspring 62-72 years **Cohort size**: 13,345 **Setting:** The Helsinki Birth Cohort study includes people born in one of the two maternity hospitals in Helsinki between 1934-44 who also attended child welfare clinics in the city.	**Exposure:** Maternal BMI prior to delivery (medical records). **Outcome:** Death, cancer, coronary heart disease, stroke, diabetes. **Ascertainment of outcome:** All-cause mortality, hospital admissions due to cardiovascular disease, coronary heart disease, stroke, or T2DM.	**Confounders:** Mother’s age and parity and for childhood and adult socio-economic status, education, and income.	In confounder-adjusted models, maternal BMI in late pregnancy is associated with increased risk of incident adverse events in offspring across 62-72 years. Each additional kg/m^2^ of maternal BMI, is associated with: Cardiovascular disease [HR: 1.03 (1.01, 1.04)] Coronary heart disease [HR: 1.03 (1.01 to 1.05)] Stroke [HR: 1.03 (1.00, 1.05)] Diabetes [HR: 1.04 (1.01, 1.07)] Hazard ratios for the obese category (compared to normal weight) are between 1.13 and 1.20 for these events Association with all-cause mortality, cancer incidence, and cancer death were not statistically significant: All-cause death [HR:1.012 (0.997, 1.028)] Cancer death [HR: 1.013 (0.983, 1.044)] Cancer incidence [HR: 1.017 (0.998, 1.036)]	Moderate
**Razaz et al., 2020** (49), Sweden **Population:** Adult offspring 1-25 years **Cohort size:** 2,230,115 **Setting:** Births recorded in the Swedish Medical Birth Register between Jan 1992-Dec 2016.	**Exposure:** MppBMI (measured at first antenatal visit, before 14 weeks in 90%). **Outcome:** Diagnosis of any cardiovascular disease **Ascertainment of outcome:** from national patient register or cause of death register.	**Confounders:** Maternal age, parity, mother’s country of origin, education level, cohabitation with partner, height, smoking status, maternal and paternal cardiovascular diseases, infant’s sex, year of delivery.	In confounder-adjusted models, early pregnancy obesity is associated with increased risk of incident adverse events in offspring over median follow up of 11.9 years: Combined cardiovascular disease obesity grade II (BMI 35 to 39.9 kg/m^2^)- [HR: 1.84 (1.36, 2.4)] obesity grade III (BMI ≥40 kg/m^2^)- [HR: 2.51 (1.60, 3.92)] Ischaemic heart disease Obesity grade II (BMI 35 to 39.9 kg/m^2^)- [HR: 2.39 (1.05, 5.47)] Heart failure Overweight (BMI 25 to 29.9 kg/m^2^)- [HR: 1.49 (1.09, 2.03)] Obesity grade II (BMI 35 to 39.9 kg/m^2^)- [HR: 3.69 (1.88, 7.27)] Cerebrovascular diseases Obesity grade III (BMI ≥40 kg/m^2^)- [HR: 2.45 (1.51, 3.97)]	Moderate

BMI, body mass index; HR, hazard ratio; MppBMI, Maternal pre-pregnancy body mass index; T2DM, Type 2 diabetes mellitus.

### Risk of Bias Assessment

The studies were evaluated for their risk of bias using the ROBINS-I tool. 16 studies were appraised to be of moderate risk of bias, while 5 were of severe risk and 5 of critical risk. One study did not have enough information available to come to a bias assessment. No studies were at a low risk of bias. The results of the bias assessment are displayed in **Supplementary Table 2** and summarised in **Tables 3**–**7**.

### Neonatal Congenital Heart Disease

Several studies examined the link between maternal obesity and neonatal CHD. Brite et al. (24) conducted a retrospective electronic records review of maternal and offspring records of 121,815 deliveries across 19 hospitals included in the Consortium on Safe Labour cohort study. Odds of any offspring CHD ascertained from hospital discharge records are reported for different pre-pregnancy BMI categories (overweight, obese, and morbidly obese) compared to normal weight mothers as defined by WHO categories. Additionally, odds of disease-specific CHDs were considered in mothers with obesity (BMI ≥30kg/m^2^) compared to mothers with BMI<30kg/m^2^. Models were adjusted for study site, age, race, insurance status, and maternal smoking. A further analysis on a subset of 5,131 pairs was also adjusted for maternal glucose tolerance. The sample included 1,388 (1%) cases of CHD. The authors report a dose-dependent increase in odds of any CHD across increasing BMI categories. Compared to normal weight mothers, mothers with morbid obesity (BMI ≥40kg/m^2^) were at highest risk of having a child with CHD [OR 1.36 (1.03-1.78)], followed by mothers with obesity [OR 1.25 (1.08-1.45)], and mothers who were overweight [OR 1.18 (1.03-1.34)]. Compared to mothers without obesity, those with BMI ≥30kg/m^2^ had significantly greater odds of having an infant with conotruncal defects [OR 1.33 (1.03–1.72)], atrial septal defects [OR 1.22 (1.04–1.43)], or ventricular septal defects [OR 1.38 (1.06–1.79)]. In the subset of 5,131 pairs adjusted for maternal glucose tolerance, these results remained consistent.

Madsen et al. (26) report a large population-based case-control study evaluating the relationship between maternal obesity and offspring CHD using linked birth-hospital discharge records between 1992-2007. The authors considered any CHD recorded within three years of birth. The sample comprised 14,142 infants with CHD and 141,420 comparators without CHD selected at random and matched on year of delivery. In models adjusted for gestational diabetes, infants with CHD were more likely to have a mother with obesity [OR 1.22, (1.15–1.30)]. Consistent with the observations by Brite et al. (24), Madsen et al. (26) also report a dose-response relationship between increasing BMI and higher risk of offspring CHD. They also report association of higher BMI with greater risk of specific CHDs, namely left [OR 1.27 (1.02–1.59)] and right [OR 1.43 (1.20–1.69)] ventricular outflow tract defects, and hypoplastic left heart syndrome [OR 1.86, 95% (1.13–3.05)]. In contrast to Brite et al. (24), they report no statistically significant association with conotruncal defects [OR 1.04 (0.82–1.33)]. These studies have several strengths, both making use of large, standardised health records to ascertain maternal BMI and offspring health outcomes. However, notably, Brite et al. (24) report missing BMI data for 77,000 women, and noted a higher prevalence of CHD in women with missing BMI data. This suggests not-at-random missingness in a manner relevant to the outcome of interest and may thus be a source of systematic bias. Similarly, Madsen et al. (26) used self-reported BMI for women who had missing physical measurements of BMI, potentially introducing recall/reporting bias in ascertainment of the exposure. Furthermore, neither study considered key modifying variables, such as exposure to teratogens, folic acid intake, and maternal co-morbidities other than gestational diabetes, which may represent important sources of confounding.

A small case control study by Alvarado-Terrones et al. (22), investigating maternal factors associated with incidence of total anomalous pulmonary venous connection (TAPVC) presented findings consistent with these observations. This study included 55 mother-child cases with isolated TAPVC and 152 healthy mother-child cases. Both groups had no maternal history of addiction, pre-eclampsia, type 1 diabetes, type 2 diabetes, or GDM. They report no difference in rates of family history, addiction, folic acid supplementation, or alcoholism in cases vs controls. The authors report that compared to normal weight mothers, those with pre-pregnancy BMI in the overweight and obese categories had significantly higher risk of infant TAPVC [OR 1.9 (0.9-3.8); OR 3.7 (1.5-9.5), respectively].

Complementary to these findings, Tang et al. (23) report association of elevated MppBMI with increased risk of obstructive heart defects (OHDs). This study identified several infant and maternal single nucleotide polymorphisms (SNPs) that interacted with maternal obesity to increase the risk of OHDs, some of which were protective in normal weight women (23) but not in those with obesity. These findings suggest important genetic evidence supporting the association between maternal BMI and infant CHD.

Several studies have failed to reproduce the observed associations between maternal BMI and infant CHD risk. Ghaderian et al. (25) present a retrospective case-control study of 164 CHD consecutive cases of infants referred to their paediatric unit and 158 infants without major malformations and their mothers. Infants with syndromic CHD, genetic syndrome, or chromosomal defects were excluded, as were mothers with gestational or pre-pregnancy diabetes. Maternal BMI was extracted from medical records and categorised into WHO obesity groups. In contrast with previously mentioned studied (22, 24, 26), Ghaderian et al. (25) report no statistically significant association between maternal obesity and infant CHD. It is possible that by more careful exclusion of genetic causes of CHD and mothers with history of diabetes, Ghaderian et al. (25) were able to elucidate the true nature of the maternal obesity-CHD relationship isolated from these confounding factors. However, the authors may also have been underpowered to detect an association with obesity due to the small number of mothers in the obesity categories (n=23 cases, 14%) as indicated by the wide confidence intervals reported by these authors (**Table 3**). There is also ambiguity around the timing of BMI measurement, without explicit reporting that the measures were taken before or during pregnancy.

Dolk et al. (27) also present a case control study of babies diagnosed with CHD before 6 months of age, excluding those with genetic syndromes and children born to mothers with pre-gestational diabetes. In a different approach to other studies, they also included stillborn babies with prenatal diagnosis of CHD, mitigating the effect of survivor bias. This is an important consideration, as CHD may result in intra-uterine death and maternal obesity is also associated with an increased risk of stillbirth. Therefore, in excluding stillborn babies, cases of CHD may be missed, underestimating the effect of MppBMI on CHD. In the study by Dolk et al. (27) cases were ascertained by paediatric cardiologists based on clinical records and International Paediatric Cardiology Code criteria. Controls were babies without CHD recruited from routine maternity outpatient clinics. BMI data was extracted from maternity health records. Mothers with pre-gestational diabetes were excluded. Similar to Ghaderian et al. (25), Dolk et al. (27) report no significant association between maternal BMI category and infant CHD in crude or multivariable models (adjusted for: maternal age, previous pregnancy, maternal education, socioeconomic deprivation, diet).

In a further study, Kaplinski et al. (28) investigated the association between genetically determined maternal obesity and offspring conotruncal defects in 466 patients identified *via* Children’s Hospital of Philadelphia (1992-2010) and the Paediatric Cardiac Genomic Consortium (2010-2012). This study reported no significant association (in contrast to Brite et al. (24) and Madsen et al. (26)), suggesting that the relationship may not be causal (28). However, only the genetic risk of obesity was assessed, not the actual presence of maternal pre-pregnancy obesity phenotype. Since obesity develops through a complex interaction of genetic and lifestyle factors, genetic non-compliance may be an issue in this study. Nevertheless, these studies highlight the need to consider the role of the genetic risk of obesity as well as BMI itself.

### Neonatal Cardiometabolic Parameters

Lemas et al. (29) recruited 753 mothers and infants from the Healthy Heart Study and 1,012 controls (**Table 4**). The exposure of interest was MppBMI. The outcomes considered were neonatal cardio-metabolic markers including cord blood glucose, insulin, glucose-insulin ratio, total and high-density lipoprotein cholesterol (HDL-c), TG, free fatty acids, and leptin (29). The study found that MppBMI was positively associated with cord-blood insulin and glucose, and negatively associated with HDL-c (29). Adjustment for neonatal adiposity and foetal growth rendered the association with insulin levels non-significant, suggesting the effects may be partially mediated by neonatal adiposity. This study adjusted for multiple potential confounders relating to health and environmental status of mother and child. A potential limitation is measurement of MppBMI, which was obtained from medical records in 90% and self-report in 10%; this approach may introduce erroneous and non-standardised reports as well as recall and information bias.

### Childhood Cardiometabolic Parameters

A total of 12 studies investigated the association between maternal BMI and cardiometabolic parameters during childhood (**Table 5**). The age range was 2-18 years. The papers studied a range of different outcomes split into six domains: childhood anthropometrics, BP, insulin levels, lipids, cardiovascular phenotypic measures.

### Childhood Anthropometrics

Nine studies report positive associations between MppBMI and offspring childhood anthropometrics (30, 31, 33, 35–38, 40, 41). Of these, three studies had critical and two had severe risk of bias, predominantly due to confounding and selection bias (**Table 5** and **Supplementary Table 2**).

Filler et al. (34) conducted a study in children from emergency departments and clinical hospital appointments, and found that in confounder-adjusted models, higher MppBMI was associated with greater BMI, SBP, HDL-c, insulin, and all body fat measures in the offspring at 6 years. However, recruitment from hospital clinics may have introduced selection bias (34). As the children included in the study were in hospital for a medical problem, they are unlikely to be representative of the general population. Furthermore, disease processes for which the children are hospitalised can have independent effects on BMI and cardiometabolic markers which may importantly influence associations in this sample. Therefore, associations observed by Filler et al. (34) may not be widely generalisable. Techur-Pedro’s doctoral thesis (35), includes a retrospective longitudinal cohort study of live births registered on the Palau Ministry of Health Maternal and Child Health Birth Registry. This study similarly reports that among children aged 4-6 years, the risk of overweight or obesity was 2.57-times higher among those born to mothers with pre-pregnancy obesity compared to children born to mothers with normal MppBMI. However, this study had a 72% attrition rate at offspring follow-up, severely limiting the internal validity of the study due to attrition bias (35).

Despite shortcomings in several of the studies in this category, there is broad consistency of findings with other, more methodologically robust studies. Perng et al. (37) report a prospective cohort of pregnant women and their children in the USA. They studied 1,090 mother-child pairs and measured offspring overall and central adiposity at 6-10 years, along with blood metabolic parameters. The study found that every 5kg/m^2^ increase in MppBMI was associated with 0.92kg higher total fat, 0.27 BMI z-score and 0.39kg trunk fat in the offspring (37). An important strength of this study was its adjustment for offspring lifestyle factors during childhood, including diet and television watching habits, as well as paternal BMI. Having a parent with obesity means that, beyond genetic factors, the child is more likely to grow up in an obesogenic environment, with unhealthy diet and exercise habits. Offspring lifestyle must be considered in studies in this area to ensure any association is not due to the lifestyle impact of having a parent with obesity. No other studies adjusted for these variables, making confounding bias due to these factors a major issue in all other studies.

Across these studies, a range of different methods were used to assess offspring anthropometrics. BMI is a useful measure of obesity because it is easy to calculate and correlates well with subcutaneous fat mass. However, this correlation is not true of all body types and some studies suggest that measures of visceral adiposity are more predictive of adverse cardiovascular complications of obesity. It was therefore valuable that several studies measured additional markers of offspring obesity and visceral adiposity (**Table 5**). In summary, published studies provide consistent evidence that MppBMI is positively associated with offspring BMI during childhood, with higher BMI categories conferring an increased risk.

### Childhood Blood Pressure

Six studies measured the impact of MppBMI on offspring BP. Five studies found that MppBMI was significantly associated with higher BP in the offspring (30, 33, 34, 37, 38) (**Table 5**). Perng et al. (37) found a positive association between MppBMI and higher offspring systolic BP. A similar association was found by Gaillard et al. (33) who used a prospective cohort study to assess the BMI of 5,908 mothers throughout pregnancy and followed up their offspring at 6 years. Higher weight in early pregnancy was associated with higher childhood systolic BP, although this was attenuated by adjustment for the child’s BMI, suggesting partial mediation by this variable (33). However, this study is limited by the fact that MppBMI was ascertained from self-report (33).

Veena et al. (31) recruited 504 pregnant women without gestational diabetes at their booking appointment and followed-up their offspring at 9.5 years, measuring offspring BMI, BP and insulin levels, as well as paternal BMI at five years. They report no association between maternal obesity during pregnancy and offspring BP after accounting for childhood BMI (**Table 5**). However, in this study maternal BMI was measured at 30 weeks’ gestation, which could reflect GWG, as opposed to MppBMI. Indeed, Gaillard et al. (33) found only early, not mid or late, pregnancy BMI to be positively associated with offspring BP. Therefore, the measurement of maternal BMI at such a late stage in pregnancy may have missed any correlation with offspring BP and could have contributed to the contradictory result to other studies in this section. Although gestational weight gain does correlate with maternal BMI, it is a proxy estimate and introduces measurement error and noise into the measurement, potentially diluting the effects of MppBMI. Finally, Wang et al. (39) investigated the interaction of maternal serum homocysteine levels and MppBMI with offspring SBP. Elevated blood homocysteine levels are known to be associated with increase CVD risk, and this study found a significant combined effect of maternal homocysteine concentration and pre-pregnancy obesity on offspring SBP, giving an example of how cardiovascular risk factors may compound offspring risk (39).

Currently available evidence on the association between MppBMI and childhood BP is high in quality and consistent, indicating a likely underlying causal effect. Gaillard et al. (33) further demonstrate attenuation (but not complete disappearance) of this relationship with adjustment for childhood BMI, suggesting a partial mediating effect of this variable. Thus overall, the current evidence suggests that maternal obesity is linked to higher offspring BP, but that this is almost completely mediated through greater obesity in childhood.

### Childhood Insulin Levels

Five studies assessed the impact of MppBMI on offspring insulin levels. All studies used fasting insulin levels as the outcome measure, and consistently found a positive correlation between MppBMI and offspring insulin levels (31, 33, 37, 38, 40). Generally, the studies were of good quality, with only the study by Gaillard et al. (33) at a serious risk of bias due to the use of self-report MppBMI.

However, it is insulin resistance, rather than absolute insulin levels, that underlies the pathophysiology of type 2 diabetes (T2DM). To assess this, Perng et al. (37) and Tan et al. (38) used the Homeostasis Model Assessment of Insulin Resistance (HOMA-IR), an accurate tool for assessing insulin resistance (42). Both studies found a positive association between MppBMI and HOMA-IR. However, in non-diabetic subjects, the HOMA-IR is less useful and fasting insulin levels are an acceptable surrogate for insulin resistance (42). Therefore, the additional use of HOMA-IR by these studies may be of limited benefit.

Tan et al. (38) also used the Whole Body Insulin Sensitivity Index (WBISI), which is of greater benefit than HOMA-IR as the use of an Oral Glucose Tolerance Test integrates both insulin sensitivity and secretion (43). When comparing the offspring of normoglycaemic mothers with overweight/obesity to the offspring of normoglycaemic normal weight mothers, Tan et al. (38) found that MppBMI was positively associated with WBISI. However, confounders such as sociodemographic factors were not considered, and self-reported MppBMI was used to measure the exposure for some participants.

Adiposity has been strongly linked to insulin resistance. Therefore, it was important that studies adjusted for current offspring anthropometrics: in the four studies that adjusted for this, the association was partially attenuated (31, 33, 38, 40). Brandt et al. (40) also showed individuals with elevated insulin levels at age eight years had a significantly higher BMI trajectory from one year onwards. In summary, MppBMI has been consistently associated with both higher levels of insulin and insulin resistance in child offspring, supporting a potential underlying causal relationship, and this association is only partially explained (and therefore mediated) by the offspring BMI.

### Childhood Lipid Profile

Four studies investigated the role of MppBMI on offspring lipid levels, although results were mixed. Gaillard et al. (33) and Tan et al. (38) found a significant association between higher MppBMI and adverse offspring lipid levels: lower HDL-c and higher low density lipoprotein-cholesterol (LDL-c) levels, though this analysis was not adjusted for by child body fat percentage or BMI and sociodemographic confounders, and consequently, the study by Gaillard et al. is at serious risk of bias from unadjusted confounders. Additionally, these findings were not supported by the study by Veena et al. (31), which found no association, although maternal BMI in this study was measured late in pregnancy at 30 weeks’ gestation. Finally, Perng et al. (37) investigated only the impact of MppBMI on triglyceride levels, reporting no significant relationship. Considering current evidence, no definitive conclusions can be made regarding the association between MppBMI and offspring lipid levels as the results are contradictory and the quality of the studies is mixed.

### Childhood Cardiac Phenotypes

Two studies used echocardiography to measure offspring cardiovascular structure and function in childhood (32, 36). In a study of 4,852 parents and children, Toemen et al. (32) found that MppBMI (self-reported) and early pregnancy BMI (measured) were associated with higher left ventricular mass and left ventricular mass index in the offspring at six years. However, these associations were attenuated to non-significance when adjusted for offspring BMI. Also, no significant association was found with relative wall thickness or fractional shortening. Similarly, Litwin et al. (36) found no association with offspring LV diastolic and systolic function, this study was at serious risk of bias as GDM was the only confounder considered.

There is a paucity of evidence in this area, which limits the conclusions that can be drawn. The results available suggest that higher MppBMI is associated with adverse cardiovascular phenotypes in childhood, but that this may be wholly related to childhood BMI. Further studies are required before definitive conclusions can be drawn in this field.

### Childhood Vascular Parameters

Two studies assessed the association between MppBMI on the offspring vasculature (30, 32). Toemen et al. (32) found MppBMI was positively associated with greater aortic root diameter of offspring measured on echocardiography. Cox et al. (30) used fundal image analysis to calculate the retinal tortuosity index, an indirect measure of hypertension, coronary heart disease and stroke risk (44). Higher MppBMI was significantly associated with an increased retinal tortuosity index, independent of BP, in the offspring at age 4-6 years (30). However, only 56% of participants were followed-up, placing the study at serious risk of bias (30). The low number of studies and heterogenous designs mean conclusions cannot be drawn about the impact of MppBMI on offspring vasculature. However, these results suggest a potential association that warrants further investigation.

### Adult Anthropometrics

Two studies measured offspring anthropometrics in adulthood (15, 45). Both studies found a positive association between MppBMI and offspring BMI (**Table 6**). Hochner et al. (15) prospectively followed-up 1,400 offspring at 32 years, using a self-report questionnaire to measure MppBMI and GWG. For every one standard deviation (SD) increase in MppBMI, offspring BMI increased by 1.8 kg/m^2^ and waist circumference (WC) increased by 3.5 cm, and similarly, greater GWG was associated with higher offspring BMI in adulthood (15). Eriksson et al. (45) followed up 2,003 offspring at 62 years and found MppBMI was positively associated with lean body mass, fat mass, and BMI of the offspring. However, this study failed to adjust for confounders, except birthweight. Maternal BMI was also measured before delivery, so measurements may reflect gestational weight gain, as opposed to MppBMI. This study is likely impacted by survival bias as those who survived until 62 years may have had a more favourable body composition than those who died. Lawrence et al. (47) measured the change in offspring BMI from 17-32 years and found a significant association between higher MppBMI and a greater interval increase in offspring BMI, even after adjustment for genetic risk scores. Overall, the results are suggestive of a positive association between MppBMI and offspring obesity in adulthood. However, the extent to which this is caused by genetic factors, environmental factors or pregnancy-related factors is difficult to isolate.

### Adult Blood Pressure

Three studies measured the impact of MppBMI on offspring BP in adulthood (15, 16, 45). Only Hochner et al. (15) found a significant positive association between MppBMI and offspring systolic and diastolic BP. However, the association was attenuated after adjustment for the current offspring BMI, indicating that offspring BMI is a full mediator of this association. Kaseva et al. (16) found no significant association between MppBMI and offspring BP at 24 years. Eriksson et al. (45) found no significant association at 62 years. In summary, MppBMI may be associated with offspring BP during adulthood, though this relationship, similar to relationships with childhood BP, may be mediated by offspring BMI.

### Adult Insulin Levels

Three studies addressed the role of MppBMI on offspring insulin levels during adulthood (15, 16, 45). Hochner et al. (15) and Kaseva et al. (16) found a significant association between MppBMI and offspring insulin levels at 32 and 24 years respectively. Kaseva et al. (16) also found a significant association with fasting glucose levels, although there is insufficient information available in the study to assess bias. The associations were attenuated to non-significance in both studies when adjusted for current offspring BMI. Eriksson et al. (45) found no association between maternal obesity and neither insulin or fasting glucose levels in offspring at 62 years but was at a serious risk of bias. While the results suggest there may be an association between MppBMI and offspring adult insulin and glucose levels, it is likely to be mediated by the offspring’s current BMI.

Two studies assessed the role of MppBMI in the risk of offspring type 2 diabetes. Lahti-Pulkkinen et al. (46) and Eriksson et al. (48) found a significant association between MppBMI and offspring diabetes risk. Lahti-Pulkkinen et al. (46) linked the birth records of 118,201 children born in Aberdeen between 1950-2011 to the Scottish register for diabetes (up to 2012). Offspring of mothers who were overweight or obese had an increased hazard of T2DM compared to normal BMI mothers (hazard ratio 3.48 and 1.39 respectively) (46).

Eriksson et al. (48) used the Helsinki Birth Cohort study to follow disease outcomes in 13,345 individuals born in Helsinki between 1934 to 1944. Maternal BMI was associated with an increased risk of diabetes (48). However, maternal BMI was measured at the end of pregnancy and did not adjust for the gestation at which the BMI was measured, failing to distinguish between GWG and MppBMI. Furthermore, Eriksson et al. identified diabetes cases through prescriptions of medication, missing diet-controlled and undiagnosed cases. This method also relies on the completeness and accuracy of medication records. Some diabetes medications can be used for other diseases, such as metformin, so there is also a risk of misclassification.

The results suggest an association between MppBMI and offspring type 2 diabetes. However, the studies have several key limitations and neither adjusted for current offspring BMI, limiting definitive conclusions.

### Adult Lipid Profile

Three studies investigated the impact on adult offspring lipid levels (15, 16, 45). Only Hochner et al. (15) found a positive association between MppBMI and offspring triglyceride levels at 32 years, but this was wholly mediated by offspring current BMI, suggesting no direct association between MppBMI and triglyceride levels. Kaseva et al. (16) and Eriksson et al. (45) similarly found no association between MppBMI and offspring lipid measures (Total Cholesterol, LDL-c, HDL-c and triglycerides), although both have key issues. Overall, it remains unclear whether there is an association between MppBMI and offspring lipid levels as existing evidence is limited to a few studies with high risk of bias.

### Adult Cardiovascular Disease

Three studies investigated the effect of maternal BMI during pregnancy on offspring CVD. All found that an elevated MppBMI was associated with increased risk of offspring hospital admission and death due to CVD (14, 48, 49) (**Table 7**).

Reynolds et al. (14) linked the Aberdeen Maternity and Neonatal Databank to national death and morbidity records for 37,709 individuals aged 34-61years. The study found that the children of mothers with obesity were more likely to be admitted to hospital for a cardiovascular event than children of mothers with a normal BMI (14).

Razaz et al. (49) used the Swedish Birth Register to follow up offspring between 1-25 years. The children of mothers who were overweight or obese were more likely to develop CVD compared to those of normal BMI mothers (49). Interestingly, Razaz et al. (49) conducted a sibling analysis, where CVD cases were matched to their siblings, in order to remove the impact of childhood environment, which the siblings likely shared. The study found that increase in maternal BMI between pregnancies was associated with an increase in offspring CVD risk (49). This finding is suggestive of a causal relationship between MppBMI and offspring CVD. However, it is important to note that despite the sibling study design, there may still be a degree of persistent environmental confounding.

Importantly, none of the studies exploring the role of MppBMI in CVD measured or adjusted for the current offspring BMI, lifestyle factors or sociodemographic factors, so residual confounding is very likely in these studies. Overall, the results suggest there is a positive association between MppBMI and risk of hospital admission and death from CVD, but this may be partly or wholly explained by confounding, and further investigations are needed to address this question.

### Main Limitations in Appraised Studies

A main limitation of existing studies is inadequate consideration of confounding. Residual confounding reduces specificity and severely limits causal conclusions. Our study question cannot ethically or practically be answered in human randomised controlled trial. Therefore, controlling for confounders needs to be a key focus of future observational studies. While controlling for all variables may not be feasible, we have identified key areas where confounding should be addressed (**Supplementary Table 3**). Furthermore, experimental studies using animal models may have a role in isolating causal relationships in this context.

Accurate and non-biased measurement of maternal obesity is clearly highly important, given that it is the exposure of interest. However, very few studies performed dedicated prospective measurement of maternal BMI as part of a research protocol. In most studies, MppBMI was ascertained from self-report questionnaires or medical records. Using self-report methods, women generally underestimate MppBMI in higher BMI categories (50). This approach may also introduce recall bias as women may forget their pre-pregnancy BMI over time. Importantly such reporting biases may differently influence self-reports of BMI in mothers of children with cardiovascular illnesses compared to those with healthy children thereby introducing systematic bias. Other studies obtained BMI from medical records. Although this is a more objective method for ascertainment of BMI, there is also potential to introduce biases given that recording of BMI is not performed using pre-defined protocols or standardised equipment.

Another important consideration is timing of maternal obesity measurement. This is clearly important given that women gain weight during pregnancy and that exposure to maternal obesity may have different consequences for the offspring depending on gestational age of exposure. Furthermore, variation in time of measurement of maternal obesity limits between study comparisons. Measuring MppBMI at a later stage in pregnancy may reflect GWG, as opposed to MppBMI. Studies should therefore aim to measure the BMI as soon as possible at a standard gestation. However, this is difficult to achieve as women may not always have a reason to attend a medical facility to have their BMI checked. Women gain minimal weight during the first trimester, indicating BMI at the 10 week booking appointment is a reliable substitute for MppBMI (51). In our review, many studies measured maternal BMI at an unknown, late, or inconsistent time.

In studies investigating the offspring during childhood or adulthood, offspring were followed up for 2-18 years and 24-72 years respectively. The risk of obesity, hypertension, insulin resistance, and hypercholesterolaemia increase with age; Lawrence et al. (47) found that the children of mothers with obesity had a higher increase in BMI between 17-32years. This makes it difficult to draw conclusions about the impact of MppBMI on cardiometabolic parameters because the effects are potentially only manifest at certain ages. To understand how the risk changes with time, measurements should be repeated at different ages. However, drop-out, time, and cost limit the feasibility of this recommendation.

A further consideration is the potential time-varying nature of exposure to maternal obesity on offspring cardiovascular risk. It is possible, for example, that the importance of maternal obesity as a driver of adverse cardiovascular health may be more important at younger rather than older ages. As an individual transitions to adulthood other environmental exposures may contribute more to the overall cardiovascular risk than maternal obesity. However, it is also reasonable to suggest, based on available evidence, that early life exposure to maternal obesity is both a direct and indirect driver of adverse cardiometabolic profile and lifestyle behaviours throughout life. Indeed, the importance of early life exposures as determinants of health through to older age is the key tenet of lifecourse epidemiology theory (52). Further research, using different approaches, is needed to determine with greater certainty the mechanisms through which maternal obesity impacts offspring cardiovascular health and to isolate its independent effect over environmental exposures in later life. Animal studies and genetic studies are likely to provide greatest insight into such mechanism as the separation of such tightly intertwined factors (maternal obesity, environment/lifestyle) using observational approaches is extremely challenging and may not be possible.

## Discussion

In this study, we systematically reviewed studies investigating the association between maternal obesity and cardiovascular health in the offspring. A large number of studies were examined with variable designs and quality, measuring the impact on CHD, cardiometabolic parameters, and CVD across a range of age groups.

### Congenital Heart Disease

Our results were suggestive of a positive association between MppBMI and CHD. Previous studies have proposed several underlying mechanisms, although no definitive cause has been found. Firstly, impaired glycaemic control increases CHD risk and individuals with obesity are more likely to experience type 2 and gestational diabetes. However, most studies in this review excluded mothers with diabetes, reducing the likelihood of this as a potential explanation of the relationship.

Folic acid supplementation decreases the risk of neural tube defects and CHD (53–55). Most studies in this review controlled for folic acid supplementation, which, in theory, would indicate folic acid deficiency does not explain the elevated risk of CHD. However, women with obesity are more likely to be deficient in folic acid despite supplementation and may require higher doses (54, 56). Therefore, future studies should measure serum folate levels, as well as supplementation. Furthermore, other teratogenic exposures were not adequately considered in existing studies.

Finally, previous studies have suggested women with obesity may be less likely to comply with antenatal screening. Similarly, increased abdominal fat may make defects less visible *via* ultrasound scan. Without a prenatal diagnosis, women with obesity may be less likely to terminate an affected pregnancy, increasing CHD rates compared to normal weight women.

### Cardiometabolic Parameters and Cardiovascular Disease

We conclude that maternal obesity during pregnancy is associated with an adverse offspring cardiometabolic profile and higher CVD risk. The next question is whether these associations are simply due to the genetic and environmental impact of having a parent with obesity, or if exposure to maternal obesity *in-utero* independently programs the foetus to have worse cardiovascular health. The first refers to the widely accepted view that obesity and CVD occur as a consequence of unhealthy lifestyle habits and genetics. A mother with obesity is more likely to eat an unhealthy diet and exercise infrequently (57). Her children are more likely to adopt these habits, increasing their risk of obesity, hypercholesterolaemia, hypertension, and insulin resistance. These risk factors would increase the offspring’s risk of CVD later in life. Furthermore, a mother with obesity may have several genetic predispositions to obesity that could further increase the offspring’s obesity risk, should these be inherited. It is possible that any associations between MppBMI and offspring cardiovascular health are simply due to the lifestyle effect of having a mother with obesity and the inheritance of obesogenic genes.

However, we found evidence that adverse cardiometabolic changes may be present in neonates of mothers with obesity. The neonatal period is not yet strongly influenced by environmental and lifestyle factors, suggesting the conditions *in-utero* may influence offspring cardiovascular health at very early ages. Furthermore, if the elevated risk was only due to lifestyle and genetic factors, paternal obesity would be expected to confer an equal risk to offspring health. Three studies in this review adjusted for paternal BMI, of these two found a significant association with an adverse offspring cardiometabolic profile even after adjustment for paternal BMI. Furthermore, Perng et al. (37) found a significant association between higher MppBMI and an adverse offspring BMI and cardiometabolic profile after adjustment for offspring television watching habits and diet during childhood. These studies suggest that offspring exposure to maternal obesity during pregnancy confers an additional risk, over that expected from lifestyle and genetic factors.

The second mechanism involves the Developmental Origins of Health and Disease (DOHaD). This was described by Barker following the observation that death rates distributions from coronary heart disease mirrored infant mortality and low birth-weight rates 70 years earlier (7). The theory postulates that exposure to less favourable conditions *in-utero* triggers physiological adaptations that help the foetus to survive (6, 7). Since these adaptations occur during critical periods of organ development, the changes are likely irreversible and impact foetal long-term development (6, 7). While these compensatory adaptations may be helpful for *in-utero* survival, the infant may be left with increased risk of developing certain non-communicable diseases later in life (6, 7). For example, the changes that help a foetus to survive conditions of poor nutrient availability *in-utero* could be maladaptive in a post-natal environment where there is sufficient food availability, increasing the offspring’s later risk of coronary heart disease. Whilst direct causal evidence in this context is lacking, several lines of evidence provide support for the hypothesis (58, 59).

The Foetal Overnutrition Hypothesis suggests that mothers with obesity are more likely to have a diet high in calories and fat, which exposes the foetus to excess nutrients *in-utero (* 12). Several observational studies have shown an association between excessive maternal fat and sugar intake and the development of childhood obesity in the offspring (12). This hypothesis is further supported by research into diabetes during pregnancy. Here, the foetus is exposed to excessive sugar, lipids and amino acids, which contribute to foetal macrosomia and an increased risk of childhood obesity (60). Furthermore, high calorie diets during pregnancy have been associated with changes in hypothalamic gene expression in animal models, which could mediate appetite and satiety in the offspring and therefore offspring obesity levels and cardiovascular risk (61).

Epigenetic changes are the second proposed underlying mechanism for the DOHaD (62). Epigenetic changes are heritable alterations in gene expression, but not the underlying genetic sequence, through DNA methylation, histone modification and RNA processes (63). The environment *in-utero* is a key period for the development of epigenetic markers; and nutrition influences this process (63). For example, offspring born during the Dutch Hunger Winter were exposed to famine conditions *in utero*. Exposed offspring were more likely to develop coronary heart disease in adulthood and at a younger age (64). Such individuals were also found to have less DNA methylation of the IGF-2 gene 60 years later compared to unexposed siblings (63). Similar epigenetic changes have been reported in animal studies for maternal obesity. In primates, a high calorie maternal diet during pregnancy was shown to cause covalent modification of histone proteins at specific sites, which altered gene expression in the offspring (65). A mouse-model found that a high-fat maternal diet during pregnancy decreased global and gene-specific DNA methylation in offspring, which altered gene expression of dopamine and opioids (66). These offspring showed an increased preference for sucrose and fat (66). Therefore, animal studies have shown exposure to pre-natal maternal obesity causes epigenetic changes that adversely influence offspring diet. However, the impact on disease risk is unclear and human studies are limited. A systematic review by Van Dijk et al. (67) found that obesity was associated with different levels of methylation at several specific sites, and some evidence that this may be reversible with weight loss programs. Liu et al. (68) measured the impact of MppBMI on CpG methylation in neonatal cord blood DNA. While one significant site was identified, the impact on gene expression and offspring long term health were not investigated, so the clinical relevance of this observation is unknown.

### Public Health Implications

The rising prevalence of obesity worldwide is a major public health concern. Obesity is a key risk factor for numerous conditions, and its rising prevalence is causing an increase in the incidence of CVD, type 2 diabetes, and several cancers. We found several associations between an elevated MppBMI and adverse offspring cardiovascular health, including an increased risk of CHD, obesity, worse cardiometabolic parameters, and greater CVD burden. The children of mothers with obesity are more likely to be obese themselves and have worse cardiovascular health. These observations highlight the importance of early life factors on later health outcomes (52). These findings are concerning for many reasons. 30% of reproductive age women are obese, which is likely to impact the health of the next generation, further perpetuating the increasing trend in the prevalence of obesity and CVD. Secondly, our results suggest the associations with offspring cardiovascular health may not be adequately explained by sharing lifestyle and genetic factors with an obese parent. Exposure to maternal obesity *in-utero* could program offspring to have worse cardiovascular health. These offspring would be high risk for developing CVD, given the likely presence of genetic, environmental, and developmental risk factors for obesity. These children could then grow up to also be mother with obesity, creating a vicious cycle, where the obesity crisis worsens with each generation.

To protect the offspring, we should address maternal BMI during and before pregnancy (59). If developmental programming is occurring, obesity prevention strategies could hypothetically reverse such programming. Meanwhile, if developmental programming is not occurring, MppBMI could be used as a screening tool to identify offspring at a high risk of cardiovascular complications. Pre- and post-natal interventions could be targeted towards such groups, reinforcing good lifestyle habits in the mother that she can pass on to her child. However, it is not enough to only address diet and lifestyle through educational interventions (69–71). Obesity is strongly influenced by health inequalities such as socioeconomic or educational status. Access to fresh healthy food is more difficult in some, predominantly poorer, areas and healthy food is often more expensive than unhealthy options, limiting its accessibility. Therefore, poverty must be addressed, and health education must have a particular focus on individuals in areas with greater socio-economic deprivation.

### Strengths and Limitations

The broad framing of the remit of this review, in terms of definition of the exposure, offspring cardiovascular health indicators, and age range covered permitted a wide-reaching assessment of the associations of maternal obesity with offspring cardiovascular health. We used a systematic and standardised approach validated by two independent investigators. However, despite a thorough and broad review of the topic, we were unable to draw definitive conclusions in several areas due to limitations of the source literature. We cannot exclude the possibility of publication bias. Finally, the heterogenous design of the studies precluded meta-analysis of the results.

## Conclusions

The world is facing an obesity epidemic. Currently, 30% of reproductive age women are obese in the UK. We conducted a systematic review of 27 papers to understand the impact of maternal obesity on offspring cardiovascular health. We conclude that higher MppBMI is associated with adverse offspring cardiovascular health throughout life. Developmental programming *in-utero* may contribute to the association, as the lifestyle impact of having an obese parent does not fully explain reported findings. However, the observational design and mixed quality of the studies limit conclusions about causality. Future studies must minimise confounding and measure MppBMI using standardised methods. In the meantime, public health strategies targeting maternal obesity are warranted to improve health of mother and child across multiple cardiovascular health areas throughout the life course.

## Data Availability Statement

The original contributions presented in the study are included in the article/**Supplementary Material**. Further inquiries can be directed to the corresponding author.

## Author Contributions

LK, SEP, and ZR-E conceptualised the idea and planned the study. LK led on running literature searches, critical appraisal of the literature, and writing of the manuscript. MA acted as a second independent searcher and assisted with critical appraisal and manuscript preparation. CM assisted with manuscript preparation. ZR-E and SEP provided key edits and the final version of the manuscript. ZR-E provided overall supervision of the work. All coauthors provided critical feedback on the final version of the manuscript and approved the submitted version.

## Funding

ZR-E recognizes the National Institute for Health Research (NIHR) Integrated Academic Training programme which supports her Academic Clinical Lectureship post and was also supported by British Heart Foundation Clinical Research Training Fellowship No. FS/17/81/33318. SEP acknowledges support from the ‘SmartHeart’ EPSRC programme grant (www.nihr.ac.uk; EP/P001009/1) and also from the CAP-AI programme, London’s first AI enabling programme focused on stimulating growth in the capital’s AI Sector. CAP-AI is led by Capital Enterprise in partnership with Barts Health NHS Trust and Digital Catapult and is funded by the European Regional Development Fund and Barts Charity. SEP has also received funding from the European Union’s Horizon 2020 research and innovation programme under grant agreement No. 825903 (euCanSHare project). CM and SN were supported by the Oxford NIHR Biomedical Research Centre (IS-BRC-1215-20008). SN was additionally supported by the Oxford British Heart Foundation Centre of Research Excellence. NCH acknowledges support from the UK Medical Research Council (MRC #405050259 and #MC_UU_12011/1), NIHR Southampton Biomedical Research Centre, University of Southampton, and University Hospital Southampton. AJL is funded by a British Heart Foundation Intermediate Research Fellowship (FS/18/3/33292). The funders provided support in the form of salaries for authors as detailed above but did not have any additional role in the study design, data collection and analysis, decision to publish, or preparation of the manuscript.

## Conflict of Interest

The authors declare that the research was conducted in the absence of any commercial or financial relationships that could be construed as a potential conflict of interest.

## Publisher’s Note

All claims expressed in this article are solely those of the authors and do not necessarily represent those of their affiliated organizations, or those of the publisher, the editors and the reviewers. Any product that may be evaluated in this article, or claim that may be made by its manufacturer, is not guaranteed or endorsed by the publisher.

## References

[1] Apovian CM . The Obesity Epidemic — Understanding the Disease and the Treatment. N Engl J Med (2016) 374:177–9. doi: 10.1056/NEJMe1514957 26760089

[2] Chandrasekaran S Neal-Perry G . Long-Term Consequences of Obesity on Female Fertility and the Health of the Offspring. Curr Opin Obstet Gynecol (2017) 29:180–7. doi: 10.1097/GCO.0000000000000364 PMC5983896 28448277

[3] Huda SS Brodie LE Sattar N . Obesity in Pregnancy: Prevalence and Metabolic Consequences. Semin Fetal Neonatal Med (2010) 15:70–6. doi: 10.1016/j.siny.2009.09.006 19896913

[4] Zaidi F . Why Mothers Die. Pract Midwife (2005) 8:24–6. 16262095

[5] Knight M Bunch K Tuffnell D Shakespeare J Kotnis R Kenyon S Kurinczuk JJ . Saving Lives, Improving Mothers’ Care - Lessons learned to inform maternity care from the UK and Ireland Confidential Enquiries into Maternal Deaths and Morbidity 2015-17. Oxford: National Perinatal Epidemiology Unit, University of Oxford 2019.

[6] Mandy M Nyirenda M . Developmental Origins of Health and Disease: The Relevance to Developing Nations. Int Health (2018) 10:66–70. doi: 10.1093/inthealth/ihy006 29528398 PMC5856182

[7] Barker DJP . The Origins of the Developmental Origins Theory. J Intern Med (2007) 261:412–7. doi: 10.1111/j.1365-2796.2007.01809.x 17444880

[8] Liu X Ding G Yang W Feng X Li Y Liu H . Maternal Body Mass Index and Risk of Congenital Heart Defects in Infants: A Dose-Response Meta-Analysis. BioMed Res Int (2019) 2019:1315796. doi: 10.1155/2019/1315796 31360700 PMC6642764

[9] Cai G Sun X Zhang L Hong Q . Association Between Maternal Body Mass Index and Congenital Heart Defects in Offspring: A Systematic Review. Am J Obstet Gynecol (2014) 211:91–117. doi: 10.1016/j.ajog.2014.03.028 24631708

[10] Heslehurst N Vieira R Akhter Z Bailey H Slack E Ngongalah L . The Association Between Maternal Body Mass Index and Child Obesity: A Systematic Review and Meta-Analysis. PloS Med (2019) 16:e1002817. doi: 10.1371/journal.pmed.1002817 31185012 PMC6559702

[11] Yu Z Han S Zhu J Sun X Ji C Guo X . Pre-Pregnancy Body Mass Index in Relation to Infant Birth Weight and Offspring Overweight/Obesity: A Systematic Review and Meta-Analysis. PloS One (2013) 8:e61627. doi: 10.1371/journal.pone.0061627 23613888 PMC3628788

[12] Gaillard R . Maternal Obesity During Pregnancy and Cardiovascular Development and Disease in the Offspring. Eur J Epidemiol (2015) 30:1141–52. doi: 10.1007/s10654-015-0085-7 PMC4684830 26377700

[13] Ludwig-Walz H Schmidt M Günther ALB Kroke A . Maternal Prepregnancy BMI or Weight and Offspring’s Blood Pressure: Systematic Review. Matern Child Nutr (2018) 14:e12561. doi: 10.1111/mcn.12561 29171150 PMC6865974

[14] Reynolds RM Allan KM Raja EA Bhattacharya S McNeill G Hannaford PC . Maternal Obesity During Pregnancy and Premature Mortality From Cardiovascular Event in Adult Offspring: Follow-Up of 1 323 275 Person Years. BMJ (2013) 347:f4539. doi: 10.1136/bmj.f4539 23943697 PMC3805484

[15] Hochner H Friedlander Y Calderon-Margalit R Meiner V Sagy Y Avgil-Tsadok M . Associations of Maternal Prepregnancy Body Mass Index and Gestational Weight Gain With Adult Offspring Cardiometabolic Risk Factors. Circulation (2012) 125:1381–9. doi: 10.1161/CIRCULATIONAHA.111.070060 PMC3332052 22344037

[16] Kaseva N Vääräsmäki M Sundvall J Matinolli H-M Sipola M Tikanmäki M . Gestational Diabetes But Not Prepregnancy Overweight Predicts for Cardiometabolic Markers in Offspring Twenty Years Later. J Clin Endocrinol Metab (2019) 104:2785–95. doi: 10.1210/jc.2018-02743 30835282

[17] Mei K Huang H Xia F Hong A Chen X Zhang C . State-Of-the-Art of Measures of the Obesogenic Environment for Children. Obes Rev (2021) 22:e13093. doi: 10.1111/OBR.13093 32725754 PMC7988549

[18] Dhana K Haines J Liu G Zhang C Wang X Field AE . Association Between Maternal Adherence to Healthy Lifestyle Practices and Risk of Obesity in Offspring: Results From Two Prospective Cohort Studies of Mother-Child Pairs in the United States. BMJ (2018) 362:k2486. doi: 10.1136/BMJ.K2486 29973352 PMC6031199

[19] PROSPERO: Inernational Prospective Register of Systematic Reviews . Available at: https://www.crd.york.ac.uk/prospero/.

[20] Moher D Liberati A Tetzlaff J Altman DG . Preferred Reporting Items for Systematic Reviews and Meta-Analyses: The PRISMA Statement. PloS Med (2009) 6:e1000097. doi: 10.1371/journal.pmed.1000097 19621072 PMC2707599

[21] Sterne JA Hernán MA Reeves BC Savović J Berkman ND Viswanathan M . ROBINS-I: A Tool for Assessing Risk of Bias in Non-Randomised Studies of Interventions. BMJ (2016) 355:i4919. doi: 10.1136/bmj.i4919 27733354 PMC5062054

[22] Alvarado-Terrones EG Perea-Cabrera M Klünder-Klünder M Segura-Stanford B Erdmenger-Orellana JR Lopez-Yañez Blanco A . Maternal Obesity as a Risk Factor for the Development of Total Anomalous Pulmonary Venous Connection in Their Offspring. Arch Med Res (2018) 49:109–13. doi: 10.1016/j.arcmed.2018.06.001 29907426

[23] Tang X Cleves MA Nick TG Li M MacLeod SL Erickson SW . Obstructive Heart Defects Associated With Candidate Genes, Maternal Obesity, and Folic Acid Supplementation. Am J Med Genet Part A (2015) 167:1231–42. doi: 10.1002/ajmg.a.36867 PMC4675451 25846410

[24] Brite J Laughon SK Troendle J Mills J . Maternal Overweight and Obesity and Risk of Congenital Heart Defects in Offspring. Int J Obes (2014) 38:878–82. doi: 10.1038/ijo.2013.244 PMC4053485 24362506

[25] Ghaderian M Emami-Moghadam A-R Khalilian M-R Riahi K Ghaedi F . Prepregnancy Maternal Weight and Body Mass Index of Children With and Without Congenital Heart Disease. Iran J Pediatr (2014) 24:313–8. PMC4276587 25562026

[26] Madsen NL Schwartz SM Lewin MB Mueller BA . Prepregnancy Body Mass Index and Congenital Heart Defects Among Offspring: A Population-Based Study. Congenit Heart Dis (2013) 8:131–41. doi: 10.1111/j.1747-0803.2012.00714.x 22967199

[27] Dolk H McCullough N Callaghan S Casey F Craig B Given J . Risk Factors for Congenital Heart Disease: The Baby Hearts Study, A Population-Based Case-Control Study. PloS One (2020) 15:e0227908. doi: 10.1371/journal.pone.0227908 32092068 PMC7039413

[28] Kaplinski M Taylor D Mitchell LE Hammond DA Goldmuntz E Agopian AJ . The Association of Elevated Maternal Genetic Risk Scores for Hypertension, Type 2 Diabetes and Obesity and Having a Child With a Congenital Heart Defect. PloS One (2019) 14:e0216477. doi: 10.1371/journal.pone.0216477 31141530 PMC6541344

[29] Lemas DJ Brinton JT Shapiro ALB Glueck DH Friedman JE Dabelea D . Associations of Maternal Weight Status Prior and During Pregnancy With Neonatal Cardiometabolic Markers at Birth: The Healthy Start Study. Int J Obes (2015) 39:1437–42. doi: 10.1038/ijo.2015.109 PMC4596750 26055075

[30] Cox B Luyten LJ Dockx Y Provost E Madhloum N De Boever P . Association Between Maternal Prepregnancy Body Mass Index and Anthropometric Parameters, Blood Pressure, and Retinal Microvasculature in Children Age 4 to 6 Years. JAMA Netw Open (2020) 3:e204662. doi: 10.1001/jamanetworkopen.2020.4662 32396192 PMC7218490

[31] Veena SR Krishnaveni GV Karat SC Osmond C Fall CH . Testing the Fetal Overnutrition Hypothesis; The Relationship of Maternal and Paternal Adiposity to Adiposity, Insulin Resistance and Cardiovascular Risk Factors in Indian Children. Public Health Nutr (2013) 16:1656–66. doi: 10.1017/S1368980012003795 PMC3622715 22895107

[32] Toemen L Gishti O van Osch-Gevers L Steegers EAP Helbing WA Felix JF . Maternal Obesity, Gestational Weight Gain and Childhood Cardiac Outcomes: Role of Childhood Body Mass Index. Int J Obes (2016) 40:1070–8. doi: 10.1038/ijo.2016.86 27143034

[33] Gaillard R Steegers EAP Franco OH Hofman A Jaddoe VWV . Maternal Weight Gain in Different Periods of Pregnancy and Childhood Cardio-Metabolic Outcomes. The Generation R Study. Int J Obes (2015) 39:677–85. doi: 10.1038/ijo.2014.175 25287752

[34] Filler G Yasin A Kesarwani P Garg AX Lindsay R Sharma AP . Big Mother or Small Baby: Which Predicts Hypertension? J Clin Hypertens (2011) 13:35–41. doi: 10.1111/j.1751-7176.2010.00366.x PMC8673311 21214720

[35] Techur-Pedro A . Maternal Prepregnancy Nutritional Status as a Key Link of Intergenerational Risk of Obesity and Chronic Disease in Childhood: University of Hawai'i at Manoa (2015). Available at: https://www.proquest.com/docview/1714412442.

[36] Litwin L Sundholm JKM Rönö K Koivusalo SB Eriksson JG Sarkola T . No Effect of Gestational Diabetes or Pre-Gestational Obesity on 6-Year Offspring Left Ventricular Function—RADIEL Study Follow-Up. Acta Diabetol (2020) 57:1463–72. doi: 10.1007/s00592-020-01571-z PMC7591425 32725413

[37] Perng W Gillman MW Mantzoros CS Oken E . A Prospective Study of Maternal Prenatal Weight and Offspring Cardiometabolic Health in Midchildhood. Ann Epidemiol (2014) 24:793–800.e1. doi: 10.1016/j.annepidem.2014.08.002 25263237 PMC4254266

[38] Tan HC Roberts J Catov J Krishnamurthy R Shypailo R Bacha F . Mother’s Pre-Pregnancy BMI is an Important Determinant of Adverse Cardiometabolic Risk in Childhood. Pediatr Diabetes (2015) 16:419–26. doi: 10.1111/pedi.12273 PMC4534350 25800542

[39] Wang H Xu BP Xu RB Walker SO Wang G . Joint Effect of Maternal Plasma Homocysteine and Prepregnancy Obesity on Child Blood Pressure: A Prospective Birth Cohort Study. Int J Obes (2017) 41:1447–53. doi: 10.1038/ijo.2017.109 PMC5585041 28465603

[40] Brandt S Moß A Lennerz B Koenig W Weyermann M Rothenbacher D . Plasma Insulin Levels in Childhood are Related to Maternal Factors - Results of the Ulm Birth Cohort Study. Pediatr Diabetes (2014) 15:453–63. doi: 10.1111/pedi.12109 24433290

[41] Sundholm JKM Litwin L Rönö K Koivusalo SB Eriksson JG Sarkola T . Maternal Obesity and Gestational Diabetes: Impact on Arterial Wall Layer Thickness and Stiffness in Early Childhood - RADIEL Study Six-Year Follow-Up. Atherosclerosis (2019) 284:237–44. doi: 10.1016/j.atherosclerosis.2019.01.037 30819513

[42] Antuna-Puente B Disse E Rabasa-Lhoret R Laville M Capeau J Bastard J-P . How Can We Measure Insulin Sensitivity/Resistance? Diabetes Metab (2011) 37:179–88. doi: 10.1016/j.diabet.2011.01.002 21435930

[43] Yeckel CW Weiss R Dziura J Taksali SE Dufour S Burgert TS . Validation of Insulin Sensitivity Indices From Oral Glucose Tolerance Test Parameters in Obese Children and Adolescents. J Clin Endocrinol Metab (2004) 89:1096–101. doi: 10.1210/jc.2003-031503 15001593

[44] Witt N Wong TY Hughes AD Chaturvedi N Klein BE Evans R . Abnormalities of Retinal Microvascular Structure and Risk of Mortality From Ischemic Heart Disease and Stroke. Hypertension (2006) 47:975–81. doi: 10.1161/01.HYP.0000216717.72048.6c 16585415

[45] Eriksson JG Sandboge S Salonen M Kajantie E Osmond C . Maternal Weight in Pregnancy and Offspring Body Composition in Late Adulthood: Findings From the Helsinki Birth Cohort Study (HBCS). Ann Med (2015) 47:94–9. doi: 10.3109/07853890.2015.1004360 25797690

[46] Lahti-Pulkkinen M Bhattacharya S Wild SH Lindsay RS Räikkönen K Norman JE . Consequences of Being Overweight or Obese During Pregnancy on Diabetes in the Offspring: A Record Linkage Study in Aberdeen, Scotland. Diabetologia (2019) 62:1412–9. doi: 10.1007/s00125-019-4891-4 PMC6647186 31214738

[47] Lawrence GM Shulman S Friedlander Y Sitlani CM Burger A Savitsky B . Associations of Maternal Pre-Pregnancy and Gestational Body Size With Offspring Longitudinal Change in BMI. Obesity (2014) 22:1165–71. doi: 10.1002/oby.20643 PMC3968220 24124160

[48] Eriksson JG Sandboge S Salonen MK Kajantie E Osmond C . Long-Term Consequences of Maternal Overweight in Pregnancy on Offspring Later Health: Findings From the Helsinki Birth Cohort Study. Ann Med (2014) 46:434–8. doi: 10.3109/07853890.2014.919728 24910160

[49] Razaz N Villamor E Muraca GM Bonamy A-KE Cnattingius S . Maternal Obesity and Risk of Cardiovascular Diseases in Offspring: A Population-Based Cohort and Sibling-Controlled Study. Lancet Diabetes Endocrinol (2020) 8:572–81. doi: 10.1016/S2213-8587(20)30151-0 32559473

[50] Stommel M Schoenborn CA . Accuracy and Usefulness of BMI Measures Based on Self-Reported Weight and Height: Findings From the NHANES & NHIS 2001-2006. BMC Public Health (2009) 9:421. doi: 10.1186/1471-2458-9-421 19922675 PMC2784464

[51] Fattah C Farah N O’Toole F Barry S Stuart B Turner MJ . Body Mass Index (BMI) in Women Booking for Antenatal Care: Comparison Between Selfreported and Digital Measurements. Eur J Obstet Gynecol Reprod Biol (2009) 144:32–4. doi: 10.1016/j.ejogrb.2009.01.015 19268433

[52] Kuh D Ben-Shlomo Y Lynch J Hallqvist J Power C . Life Course Epidemiology. J Epidemiol Community Heal (2003) 57:778–83. doi: 10.1136/JECH.57.10.778 PMC1732305 14573579

[53] Feng Y Wang S Chen R Tong X Wu Z Mo X . Maternal Folic Acid Supplementation and the Risk of Congenital Heart Defects in Offspring: A Meta-Analysis of Epidemiological Observational Studies. Sci Rep (2015) 5:8506. doi: 10.1038/srep08506 25687545 PMC4330542

[54] Zheng Z Yang T Chen L Wang L Zhang S Wang T . Increased Maternal Body Mass Index is Associated With Congenital Heart Defects: An Updated Meta-Analysis of Observational Studies. Int J Cardiol (2018) 273:112–20. doi: 10.1016/j.ijcard.2018.09.116 30293662

[55] Rasmussen SA Chu SY Kim SY Schmid CH Lau J . Maternal Obesity and Risk of Neural Tube Defects: A Metaanalysis. Am J Obstet Gynecol (2008) 198:611–9. doi: 10.1016/j.ajog.2008.04.021 18538144

[56] Mojtabai R . Body Mass Index and Serum Folate in Childbearing Age Women. Eur J Epidemiol (2004) 19:1029–36. doi: 10.1007/s10654-004-2253-z 15648596

[57] Mozaffarian D Hao T Rimm EB Willett WC Hu FB . Changes in Diet and Lifestyle and Long-Term Weight Gain in Women and Men. N Engl J Med (2011) 364:2392–404. doi: 10.1056/NEJMOA1014296 PMC3151731 21696306

[58] Barker DJP . Obesity and Early Life. Obes Rev (2007) 8:45–9. doi: 10.1111/J.1467-789X.2007.00317.X 17316301

[59] Hanson M Barker M Dodd JM Kumanyika S Norris S Steegers E . Interventions to Prevent Maternal Obesity Before Conception, During Pregnancy, and Post Partum. Lancet Diabetes Endocrinol (2017) 5:65–76. doi: 10.1016/S2213-8587(16)30108-5 27743974

[60] Fall CHD . Evidence for the Intra-Uterine Programming of Adiposity in Later Life. Ann Hum Biol (2011) 38:410–28. doi: 10.3109/03014460.2011.592513 PMC3428869 21682572

[61] Poston L . Maternal Obesity, Gestational Weight Gain and Diet as Determinants of Offspring Long Term Health. Best Pract Res Clin Endocrinol Metab (2012) 26:627–39. doi: 10.1016/j.beem.2012.03.010 22980045

[62] Gluckman PD Hanson MA Cooper C Thornburg KL . Effect of *In Utero* and Early-Life Conditions on Adult Health and Disease. N Engl J Med (2008) 359:61–73. doi: 10.1056/NEJMra0708473 18596274 PMC3923653

[63] Heijmans BT Tobi EW Stein AD Putter H Blauw GJ Susser ES . Persistent Epigenetic Differences Associated With Prenatal Exposure to Famine in Humans. Proc Natl Acad Sci U.S.A. (2008) 105:17046–9. doi: 10.1073/pnas.0806560105 PMC2579375 18955703

[64] Painter RC De Rooij SR Bossuyt PM Simmers TA Osmond C Barker DJ . Early Onset of Coronary Artery Disease After Prenatal Exposure to the Dutch Famine. Am J Clin Nutr (2006) 84:322–7. doi: 10.1093/ajcn/84.1.322 16895878

[65] Aagaard-Tillery KM Grove K Bishop J Ke X Fu Q McKnight R . Developmental Origins of Disease and Determinants of Chromatin Structure: Maternal Diet Modifies the Primate Fetal Epigenome. J Mol Endocrinol (2008) 41:91–102. doi: 10.1677/JME-08-0025 18515302 PMC2959100

[66] Vucetic Z Kimmel J Totoki K Hollenbeck E Reyes TM . Maternal High-Fat Diet Alters Methylation and Gene Expression of Dopamine and Opioid-Related Genes. Endocrinology (2010) 151:4756–64. doi: 10.1210/en.2010-0505 PMC2946145 20685869

[67] Van Dijk SJ Molloy PL Varinli H Morrison JL Muhlhausler BS Buckley M . Epigenetics and Human Obesity. Int J Obes (2015) 39:85–97. doi: 10.1038/ijo.2014.34 24566855

[68] Liu X Chen Q Tsai H-J Wang G Hong X Zhou Y . Maternal Preconception Body Mass Index and Offspring Cord Blood DNA Methylation: Exploration of Early Life Origins of Disease. Environ Mol Mutagen (2014) 55:223–30. doi: 10.1002/em.21827 PMC4547934 24243566

[69] Adams J Mytton O White M Monsivais P . Why Are Some Population Interventions for Diet and Obesity More Equitable and Effective Than Others? The Role of Individual Agency. PloS Med (2016) 13:e1001990. doi: 10.1371/JOURNAL.PMED.1001990 27046234 PMC4821622

[70] McGill R Anwar E Orton L Bromley H Lloyd-Williams F O’Flaherty M . Are Interventions to Promote Healthy Eating Equally Effective for All? Systematic Review of Socioeconomic Inequalities in Impact Health Behavior, Health Promotion and Society. BMC Public Health (2015) 15:1–15. doi: 10.1186/S12889-015-1781-7/TABLES/4 25934496 PMC4423493

[71] Beauchamp A Backholer K Magliano D Peeters A . The Effect of Obesity Prevention Interventions According to Socioeconomic Position: A Systematic Review. Obes Rev (2014) 15:541–54. doi: 10.1111/OBR.12161 24629126

