# Supplementary material for: The Impact of Maternal Obesity on Offspring Cardiovascular Health: A Systematic Literature Review
Source: Front Endocrinol (Lausanne). 2022 May 20;13:868441. doi: 10.3389/fendo.2022.868441 (PMC9164814; doi:10.3389/fendo.2022.868441)
Supplement: Supplementary file 1 [file DataSheet_1.pdf]

Supplementary Table 1. PRISMA Checklist for Systematic Reviews

Section/topic	#	Checklist item	Reported on page #
**TITLE**			
Title	1	Identify the report as a systematic review, meta-analysis, or both.	1
**ABSTRACT**			
Structured summary	2	Provide a structured summary including, as applicable: background; objectives; data sources; study eligibility criteria, participants, and interventions; study appraisal and synthesis methods; results; limitations; conclusions and implications of key findings; systematic review registration number.	2
**INTRODUCTION**			
Rationale	3	Describe the rationale for the review in the context of what is already known.	3-4
Objectives	4	Provide an explicit statement of questions being addressed with reference to participants, interventions, comparisons, outcomes, and study design (PICOS).	4, Table 1
**METHODS**			
Protocol and registration	5	Indicate if a review protocol exists, if and where it can be accessed (e.g., Web address), and, if available, provide registration information including registration number.	4
Eligibility criteria	6	Specify study characteristics (e.g., PICOS, length of follow-up) and report characteristics (e.g., years considered, language, publication status) used as criteria for eligibility, giving rationale.	Table 1
Information sources	7	Describe all information sources (e.g., databases with dates of coverage, contact with study authors to identify additional studies) in the search and date last searched.	4
Search	8	Present full electronic search strategy for at least one database, including any limits used, such that it could be repeated.	4, Table 2, Fig. 1

Study selection	9	State the process for selecting studies (i.e., screening, eligibility, included in systematic review, and, if applicable, included in the meta-analysis).	4, Table 1, Fig. 1
Data collection process	10	Describe method of data extraction from reports (e.g., piloted forms, independently, in duplicate) and any processes for obtaining and confirming data from investigators.	4, Table 1
Data items	11	List and define all variables for which data were sought (e.g., PICOS, funding sources) and any assumptions and simplifications made.	4, Table 1
Risk of bias in individual studies	12	Describe methods used for assessing risk of bias of individual studies (including specification of whether this was done at the study or outcome level), and how this information is to be used in any data synthesis.	4
Summary measures	13	State the principal summary measures (e.g., risk ratio, difference in means).	NA
Synthesis of results	14	Describe the methods of handling data and combining results of studies, if done, including measures of consistency (e.g., I^2) for each meta-analysis.	NA

**Section/topic**	**#**	**Checklist item**	**Reported on page #**
Risk of bias across studies	15	Specify any assessment of risk of bias that may affect the cumulative evidence (e.g., publication bias, selective reporting within studies).	4, Suppl. Tables 2
Additional analyses	16	Describe methods of additional analyses (e.g., sensitivity or subgroup analyses, meta-regression), if done, indicating which were pre-specified.	NA
**RESULTS**			
Study selection	17	Give numbers of studies screened, assessed for eligibility, and included in the review, with reasons for exclusions at each stage, ideally with a flow diagram.	5, Fig. 1

Study characteristics	18	For each study, present characteristics for which data were extracted (e.g., study size, PICOS, follow-up period) and provide the citations.	Tables 3-7
Risk of bias within studies	19	Present data on risk of bias of each study and, if available, any outcome level assessment (see item 12).	Suppl. Tables 2
Results of individual studies	20	For all outcomes considered (benefits or harms), present, for each study: (a) simple summary data for each intervention group (b) effect estimates and confidence intervals, ideally with a forest plot.	5-13, Tables 3-7
Synthesis of results	21	Present results of each meta-analysis done, including confidence intervals and measures of consistency.	NA
Risk of bias across studies	22	Present results of any assessment of risk of bias across studies (see Item 15).	Suppl. Tables 2
Additional analysis	23	Give results of additional analyses, if done (e.g., sensitivity or subgroup analyses, meta-regression [see Item 16]).	NA
**DISCUSSION**			
Summary of evidence	24	Summarize the main findings including the strength of evidence for each main outcome; consider their relevance to key groups (e.g., healthcare providers, users, and policy makers).	13-16
Limitations	25	Discuss limitations at study and outcome level (e.g., risk of bias), and at review-level (e.g., incomplete retrieval of identified research, reporting bias).	16
Conclusions	26	Provide a general interpretation of the results in the context of other evidence, and implications for future research.	16
**FUNDING**			
Funding	27	Describe sources of funding for the systematic review and other support (e.g., supply of data); role of funders for the systematic review.	17

Supplementary Table 2. Bias Assessment of the Observational Studies using the Robins-1 Tool

Author	Citation	Bias due to confounding	Bias in selection of participants into study	Bias in classification of interventions	Bias due to deviations from intended interventions	Bias due to missing data	Bias in measurement of outcomes	Bias in selection of reported result	Overall risk of bias
**Neonates – Congenital Heart Disease**									
Alvarado-Terrones et al. 2018	(24)								
Tang et al. 2015	(25)								
Brite et al. 2014	(22)								
Ghaderian et al. 2014	(26)								
Madsen et al. 2013	(23)								
Dolk et al. 2020	(27)								
Kaplinski et al. 2019	(28)								
**Neonates – Cardiometabolic Parameters**									
Lemas et al. 2015	(29)								

Author	Citation	Bias due to confounding	Bias in selection of participants into study	Bias in classification of interventions	Bias due to deviations from intended interventions	Bias due to missing data	Bias in measurement of outcomes	Bias in selection of reported result	Overall risk of bias
**Children – Cardiometabolic Parameters**									
Cox et al. 2020	(30)								
Veena et al. 2013	(31)								
Toemen et al. 2016	(43)								
Gaillard et al. 2015	(32)								
Filler et al. 2011	(39)								
Techur-Pedro 2015	(38)								
Litwin et al. 2020	(33)								
Perng et al. 2014	(34)								
Tan et al. 2015	(35)								
Wang et al. 2017	(40)								

Author	Citation	Bias due to confounding	Bias in selection of participants into study	Bias in classification of interventions	Bias due to deviations from intended interventions	Bias due to missing data	Bias in measurement of outcomes	Bias in selection of reported result	Overall risk of bias
Brandt et al. 2014	(36)								
Sundholm et al. 2019	(37)								
**Adults – Cardiometabolic Parameters**									
Hochner et al. 2012	(15)								
Kaseva et al. 2019	(16)								
Lahti-Pulkkinen et al. 2019	(47)								
Eriksson et al. 2015	(45)								
Lawrence et al. 2014	(46)								
**Adults – Cardiovascular Disease**									
Reynolds et al. 2013	(14)								
Eriksson et al. 2014	(48)								

Author	Citation	Bias due to confounding	Bias in selection of participants into study	Bias in classification of interventions	Bias due to deviations from intended interventions	Bias due to missing data	Bias in measurement of outcomes	Bias in selection of reported result	Overall risk of bias
Razaz et al. 2020	(49)								

Supplementary Table 2 footnote. The bias risk following appraisal using the Robins-1 tool is represented by the following colours: low risk by green, moderate risk by pale orange, severe risk by bright orange and critical risk by red. If the study had insufficient information to reach a conclusion about the bias risk, the square has been coloured grey.

Supplementary Table 3. Important Areas of Confounding

**Outcome**	**Confounder**
Congenital Heart Disease	• Teratogens increase the risk of CHD e.g. smoking and alcohol. Obese mothers may be more likely to take teratogens before or during pregnancy and may be more likely to have an offspring with a CHD as a result.<br>• Pregnancy folic acid intake reduces the risk of congenital abnormalities like neural tube defects. Obesity may affect an individual's likelihood of taking folic acid supplements during pregnancy, affecting the offspring CHD risk. Several studies also hypothesise that maternal obesity may reduce folic acid availability in utero, increasing the risk of CHD.<br>• Maternal obesity increases the risk of still-birth. Therefore, cases of CHD in the obese group would be more likely to be missed due to infant death.
Cardiovascular disease - General	• Lower socioeconomic status and education level increase obesity, smoking and cardiovascular risk. Therefore, offspring of obese mothers are likely to inherit an adverse SES from the parents and so are more likely to be obese, smoke an develop CVD as a result.<br>• Maternal obesity is associated with adverse pregnancy outcomes, such as SGA, LGA and birthweight affects CVD risk. Therefore, any association between maternal obesity and offspring CVD may be due to infant birthweight.<br>• Maternal medical conditions, particularly DM and GDM, adversely affect offspring cardiovascular risk. Obese mothers are also more likely to develop DM and GDM. Therefore, the exposure to diabetes in utero may increase the infants' risk of cardiovascular disease directly, as well as indirectly as the offspring would be at a greater risk of diabetes which increases cardiovascular disease risk.<br>• Certain genes increase the risk of obesity and hypertension. Therefore, the offspring may inherit a genetic predisposition for obesity or hypertension from the parents, meaning they themselves are more likely to be obese or hypertensive, and more likely to develop CVD as a result.
Cardiovascular disease – Children	• Offspring of obese parents would be more likely to have poor diet and exercise habits during childhood, which would influence offspring cardiovascular disease risk. This could be controlled for directly by measuring the diet and exercise levels of the offspring. Otherwise, measuring paternal BMI could extrapolate the influence of having an obese parent from the effects of exposure to maternal obesity specifically in utero.

	• Wang et al. adjusted for the Tanner stage of puberty. At different stages of puberty, the body has different levels of insulin resistance and body composition, which could confound measurements of cardiometabolic factors.
Cardiovascular disease - Adults	• Offspring of obese parents may be more likely to have poor diet and exercise habits throughout life, which would influence cardiovascular disease risk. This could be controlled for directly by measuring the diet, exercise and smoking habits of the offspring. Otherwise, measuring paternal BMI could extrapolate the influence of having an obese parent from the effects of exposure to maternal obesity specifically in utero.

Supplementary Table 3 footnote. BMI: body mass index, CHD: congenital heart disease, CVD: cardiovascular disease, DM: diabetes mellitus, GDM: gestational diabetes mellitus, SGA: small for gestational age, LGA: large for gestational age.
